# Reasons for encounter and recommended interventions in an interprofessional integrative oncology counseling program in outpatient care: a mixed-methods analysis of 1,693 consultation letters

**DOI:** 10.3389/fmed.2026.1907654

**Published:** 2026-10-07

**Authors:** Andreas Schmitt, Regina Stolz, Holger Mauch, Franziska Mergel, Barbara Grün, Mirjam Krug, Cornelia Mahler, Stefanie Joos, Jan Valentini

**Affiliations:** 1 Institute of General Practice and Interprofessional Care, University Hospital and Faculty of Medicine Tuebingen, Tuebingen, Germany; 2 Department of Gynecology and Obstetrics, SLK Clinics Heilbronn, Heilbronn, Germany; 3 Department of Medical Oncology, University Hospital and Faculty of Medicine Heidelberg, Heidelberg, Germany; 4 Department of Nursing Science, Institute of Health Sciences, University Hospital and Faculty of Medicine Tuebingen, Tuebingen, Germany

**Keywords:** CIH, complementary and integrative healthcare, complementary medicine, integrative medicine, integrative oncology, interprofessional counseling, naturopathic nursing intervention

## Abstract

**Introduction:**

Complementary and integrative healthcare (CIH) approaches play an increasingly important role in oncology care, involving a wide range of healthcare professionals and therapeutic methods. While patient demand for CIH and the evidence base for individual interventions have grown considerably, the implementation of structured counseling services for the effective and safe use of CIH remains limited. To address this gap, a controlled, multicenter implementation study was conducted at four Comprehensive Cancer Centers (CCC) in Germany to establish an outpatient, interprofessional, evidence-based counseling service for CIH (CCC-Integrativ). This mixed-methods study investigates the specific reasons for encounter (RfE) and the recommended interventions during these consultations.

**Methods:**

Patients enrolled in the CCC-Integrativ study received three CIH consultations within a three-month period, the first of which was conducted jointly by specifically trained physicians and nurses. After each session, a consultation letter was prepared summarizing the main patient RfE and recommendations. All consultation letters were analyzed using qualitative content analysis, and the results were subsequently analyzed quantitatively.

**Results:**

A total of 1,693 consultation letters from 603 oncology patients were analyzed. The most frequent reasons for encounter included general weakness and fatigue (56%), chemotherapy-induced peripheral neuropathy (40%), sleep disturbances (37%), and general pain (36%). Nearly all patients received counseling on lifestyle modifications, including nutrition (95%), physical activity (93%), and stress management (81%). Additionally, an average of 15 specific CIH interventions were recommended per patient, encompassing naturopathic nursing interventions (97%), herbal medicines and teas (79%), acupressure (79%), and mindfulness-based movement therapies (44%).

**Discussion:**

Interprofessional outpatient counseling on CIH in oncology addresses a wide spectrum of patient RfE, provides guidance on lifestyle modifications, and offers specific, individualized CIH interventions to help patients manage burdensome symptoms. These findings suggest that interprofessional integrative counseling services can play a crucial role in outpatient oncology care by providing a holistic approach tailored to individual needs. Furthermore, structured integrative oncology counseling may serve a valuable navigational role within outpatient care settings.

## 1 Introduction

Integrative oncology (IO) is defined as a “patient-centered, evidence-informed field of cancer care” that incorporates various complementary and integrative healthcare (CIH) approaches from different traditions, including “mind–body interventions, natural products, and/or lifestyle modifications alongside conventional cancer treatments” (1). The use of CIH among oncology patients in Germany has increased substantially in recent decades, with reported prevalence rates ranging from 40 to 89.4%, depending on the study design and the patient population examined (2–4). Similar rates are found in other countries, e.g., the Netherlands (40%) (5), Switzerland (56%) (6) and the USA (87.9%) (7).

Evidence supporting the effectiveness of individual CIH interventions has likewise expanded, resulting in the development and recommendation of several national and international guidelines like the German “S3-Guideline Complementary Medicine in the Treatment of Oncological Patients” for cancer in general (8), the European “5th ESO-ESMO International Consensus Guidelines for Advanced Breast Cancer” (9), the American “ASCO Endorsement of the SIO Clinical Practice Guideline” for breast cancer (10), and several American SIO guidelines for specific symptoms among cancer patients such as depression (11), fatigue (12), and others. Within this context, lifestyle modifications represent a particularly important component. Physical activity (PA) has been shown to alleviate a variety of cancer-related symptoms, including cancer-related fatigue (13), anxiety, depression, and reduced quality of life (14), as well as chemotherapy-induced peripheral neuropathy (CIPN) (15). Furthermore, PA may improve tumor response and treatment adherence during chemotherapy (12), and it has been associated with improved outcomes in both the primary (16) and secondary prevention of cancer (17, 18). A substantial body of evidence also exists regarding the role of nutrition in cancer prevention and management. Dietary factors that have been shown to influence cancer risk and outcomes include meat consumption, which increases the risk of colorectal cancer, for example (19), and vegetarian dietary patterns, which decrease the risk of a number of cancers (20). Fiber intake also decreases the risk of certain cancers, such as breast cancer (21, 22). In addition, the early identification and treatment of nutritional deficiencies represents a key component of nutritional management in oncology (23). Regarding stress management, positive evidence has been reported for several mind-body and relaxation techniques, such as Mindfulness-Based Stress Reduction (MBSR) (24), Yoga (25, 26), Tai Chi, and Qigong (27).

In addition to lifestyle modifications, a wide range of CIH interventions may help cancer patients manage their symptoms. Numerous systematic reviews have examined the efficacy of acupuncture and, to a lesser extent, acupressure. These studies suggest potential benefits for a variety of cancer-related ailments, including sleep disturbances (28), pain (29), cancer-related fatigue (30), and aromatase inhibitor–associated joint pain (31), among other symptom domains (32, 33). In the field of phytotherapy, several substances have demonstrated positive evidence for various complaints, including *Boswellia serrata*, *Cimicifuga racemosa, Cannabis sativa, Panax ginseng, Zingiber officinale*, and *Viscum album* (8). In contrast, naturopathic nursing interventions (NNI) have thus far primarily been recommended based on empirical experience and systematically developed expert consensus (34–37).

In addition to physicians, nursing professionals play an essential role in the primary care of oncology patients within integrative oncology settings (38–43). Nurses are frequently consulted regarding naturopathic or complementary care interventions aimed at alleviating treatment-related side effects and preventing complications (44). They directly implement various measures, including specialized skin and mucosal care, rubs, oil and ointment applications, partial or full baths, inhalations, moist compresses and wraps, cold and heat therapies, and therapeutic washes (45). Oncology nursing consultations are widely advocated, particularly by the medical profession (46). Evidence suggests that these consultations can address unmet physical, psychological, and social needs, thereby enhancing patient-centered, effective care (47), and improving the provision of patient information (48). Consequently, substantial advancements in patient satisfaction, quality of life, self-efficacy, and health-related behaviors have been documented (49). Within this framework, interprofessional collaboration (IPC) has increasingly gained attention as a cornerstone of integrative oncology care (37, 50–52).

Despite the widespread demand for, utilization of, and growing evidence base supporting CIH interventions, notable risks persist. These primarily concern biological therapies and their potential interactions with antitumor agents (53–55), as well as scenarios where patients reject conventional oncological treatments in favor of unproven approaches (56). This challenge is further compounded by the fact that up to 80% of patients do not disclose their CIH use to healthcare providers (57), while many clinicians report limited competence or confidence in this domain (42, 58–60). Consequently, a critical shortage of structured, evidence-based, and interprofessionally delivered outpatient CIH counseling persists (60). Furthermore, it remains unclear to what extent the broad spectrum of available interventions can be systematically integrated into clinical workflows and which specific RfE can be addressed.

To address this gap, an outpatient, evidence-based interprofessional counseling program focusing on CIH was implemented at four Comprehensive Cancer Centers (CCC), the University Clinics of Tübingen, Heidelberg, Freiburg, and Ulm. in Baden-Württemberg, Germany, within the “CCC-Integrativ” study (61). The present study is a subanalysis of the CCC-Integrativ study; therefore, we will briefly describe the latter, including its inclusion criteria and interventions. Eligibility criteria included a confirmed cancer diagnosis with initial diagnosis, recurrence, or disease progression within the previous six months, ongoing oncological treatment at one of the participating CCCs, an expressed need for CIH, and a minimum age of 18 years. Patients generally received three consultations from an interprofessional team (consisting of a physician and a nurse) over a three-month period. The first consultation lasted approximately 60 min and was conducted jointly by a physician and a nurse. Subsequent consultations could be conducted by either profession, depending on patient needs and availability, and lasted approximately 30 min each. Following each consultation in the CCC-Integrativ study, the topics discussed, the reasons for encounter (RfE), and the recommended interventions were documented in consultation letters.

The counseling team comprised experienced nursing staff with advanced professional training in areas such as oncology nursing, palliative care, external applications, aromatherapy, and anthroposophical nursing. The medical staff consisted of experts from various disciplines (including hemato-oncology, gynecology, general practice, and visceral surgery) with specialized training in traditional Chinese medicine (TCM), acupuncture, naturopathy, anthroposophical medicine, and phytotherapy. For the CCC-Integrativ study, the staff completed a specifically developed training program in Integrative Oncology (IO) and utilized customized consultation guidelines containing evidence-based information on a wide range of CIH interventions for various symptoms (61).

The CIH interventions used in the CCC-Integrativ study were developed through a structured, modified Delphi process analogous to that described by Stolz et al. (34) and were compiled by a 20-member expert team from the four CCC-Integrativ sites following a defined procedure (61, 62). First, a literature review focusing on current oncology guidelines and peer-reviewed publications was conducted to gather external evidence. Additionally, internal evidence regarding CIH interventions was systematically collected from clinical experts. All identified interventions were then evaluated based on effectiveness, safety, applicability, and cost considerations. When safety, applicability, and cost-effectiveness were positively assessed through the literature review, the effectiveness of the interventions was rated on an ascending 5-point Likert scale. To be included in the NNI catalog, an intervention had to achieve an overall score of at least three out of five points at a minimum of two of the four study sites. For medications, a more stringent threshold of four out of five points was required. This higher criterion was established to account for the increased safety risks and regulatory requirements associated with pharmacological interventions, such as drug interactions and adverse effects, as well as the study's primary focus on patient activation. Within the specifically developed training program in Integrative Oncology, the counseling teams received comprehensive training on the potential side effects and interactions between phytotherapeutics and conventional cancer treatments. The medicines and teas (MT) recommended in the study were selected with a strong emphasis on patient safety. For instance, green tea leaves were only recommended in the form of tea, rather than concentrated extracts such as capsules. Importantly, no severe adverse events (SAEs) relating to counseling or the recommended interventions were reported throughout the CCC-Integrativ study (51).

Publications arising from the CCC-Integrativ study have highlighted the effectiveness of these interprofessional consultations with regard to patient activation (51), symptom management, and patient-centered care (63), as well as the quality of interprofessional collaboration, responsiveness to patient needs, and resource allocation (64). However, comprehensive analyses examining the specific RfE and recommended interventions of the CCC-Integrativ study are still lacking, leaving the concrete situation regarding CIH in outpatient oncological care unaddressed. This study aims to bridge this research gap by analyzing further research questions: What are the most frequent reasons for encounter (RfE) and corresponding CIH recommendations documented within the CCC-Integrativ outpatient counseling program? How do these recommendations correspond to existing evidence and guidelines, and where do gaps in the evidence remain? How are specific interventions tailored to the most burdensome and prevalent symptoms identified as RfE in supportive oncology? Are there differences according to the severity/stage of the cancer diagnosis?

## 2 Materials and methods

### 2.1 Study design and setting

This study employed a mixed-methods design combining qualitative content analysis with a subsequent quantitative analysis of coded categories. The analysis was based on consultation letters generated within the CCC-Integrativ study, a controlled multicenter implementation study evaluating an interprofessional outpatient counseling program for CIH in oncology at four Comprehensive Cancer Centers (CCC) in Baden-Württemberg, Germany (51).

### 2.2 Data source: consultation letters

The participants in the study received three interprofessional counseling sessions on specific CIH interventions. After each consultation, patients received a consultation letter summarizing the content of the encounter. Across all study sites, these letters followed a standardized structure: first, the RfE were documented, followed by a description of the lifestyle modifications discussed during the consultation, and finally a list of the recommended CIH interventions, including referrals to further services where necessary. The letters were written using an interprofessional approach, with contributions from both nurses and physicians. Responsibility for drafting the initial version alternated between members of the counseling team, while the second professional reviewed and supplemented the document. Such an interprofessional documentation process was unprecedented in the context of oncological counseling, requiring the integration of dedicated electronic health record access rights for nursing staff within the hospital information system.

The consultation letters were addressed not only to the patients themselves but also to the healthcare professionals involved in their ongoing care, particularly oncologists, general practitioners, and therapists in both outpatient and inpatient settings. They served as a key source of information regarding the consultation content to ensure a seamless flow of information. Additionally, the letters were compiled and made accessible through the university hospital's electronic health record system. This ensured that the interprofessional CCC-Integrativ consultation service was granted the same level of transparency and institutional visibility as the documentation of other specialist departments involved in the patients’ oncological care.

These documents served as the primary data source for the present analysis. Letters were included for all patients who had completed the intervention phase – consisting of the three counseling sessions – and who had completed the questionnaire at time point T2 (end of intervention). Conversely, the documentation of patients who did not meet these criteria was excluded from the analysis as dropouts. This inclusion/exclusion criterion was selected to align with the methodology of the CCC-Integrativ study (51). The analyzed letters covered the period from February 2021 to September 2022. The section relevant for analysis, namely the documentation of the consultation itself, comprised approximately 0.5 to 1.5 pages per encounter. Data extraction at the four study sites was linked to the pseudonymized study ID assigned to each participant, which was used consistently across all CCC-Integrativ sites. The analyzed RfE and CIH interventions were collected from all three consultations and were not differentiated for the individual consultations.

### 2.3 Qualitative analysis

The consultation letters were analyzed using qualitative content analysis according to Mayring (65). This method allows for a systematic, rule-guided analysis of textual data and enables the integration of deductive and inductive category development.

The analysis followed a deductive–inductive approach. Prior to the coding process, a preliminary coding framework was developed deductively based on the counseling manual of the CCC-Integrativ study, existing literature on integrative oncology, and the standardized structure of the consultation letters. To facilitate a systematic analysis, RfE were classified according to the “Reason for Encounter” component of the International Classification of Primary Care (ICPC-3) (66) into 12 overarching ICPC-3 categories comprising 48 specific RfE. Three predefined categories covered lifestyle modifications (nutrition, physical activity, and relaxation/stress management). Additionally, 155 categories were defined for the recommended CIH interventions, which were subdivided into non-pharmacological interventions (NPI), naturopathic nursing interventions (NNI), and medicines and teas (MT). To ensure consistency in coding, this initial category system was reviewed by two researchers (AS and JV) and a comprehensive coding guide was developed (Supplementary 1).

The consultation letters were analyzed and coded by the respective counseling staff at each study site using the developed category system. This research team comprised experts from the counseling teams across the four CCC sites, including nurses with backgrounds in oncology and integrative nursing, and physicians with backgrounds in oncology, gynecology, visceral surgery, and general practice. Due to the German General Data Protection Regulation (GDPR), it was not possible to code all consultation letters at a single study site. To ensure optimal standardization, the research team performing the coding was trained in the final category system and coding rules during specific online sessions. During the coding process, the category system was iteratively refined. Whenever relevant content could not be assigned to an existing category, additional categories were developed inductively in accordance with Mayring's procedures for category formation. Definitions for all categories were documented and continuously refined throughout the coding process. Consequently, the category system expanded to include 67 categories for RfE, three for lifestyle modifications, and 252 for recommended CIH interventions. Discrepancies were discussed within the research teams until a consensus was reached, after which the category system was further refined and finalized. A list of all 67 categories for RfE and the 252 recommended interventions can be found in Supplementary 2. The finalized coding framework was subsequently applied to the entire dataset. Regular meetings with the research teams from the different study sites were conducted to discuss uncertainties in category assignment and to ensure the consistent application of the coding rules.

### 2.4 Quantitative analysis

Following the completion of the qualitative coding process, the coded data and categories were imported into Microsoft Excel as a relational database and analyzed quantitatively using descriptive statistics. For each category, the frequency and proportion of consultation letters containing the respective category were calculated. Various queries enabled different groupings based on reasons for encounter, lifestyle modifications, recommended interventions, substances, study sites, diagnoses, and the interrelationships between these variables. Figure 1 illustrates the workflow of the analysis. The detailed code sheet and the coding guide are available in the supplementary material (Figure 1; Supplementary 1 and 2).

**Figure 1:**
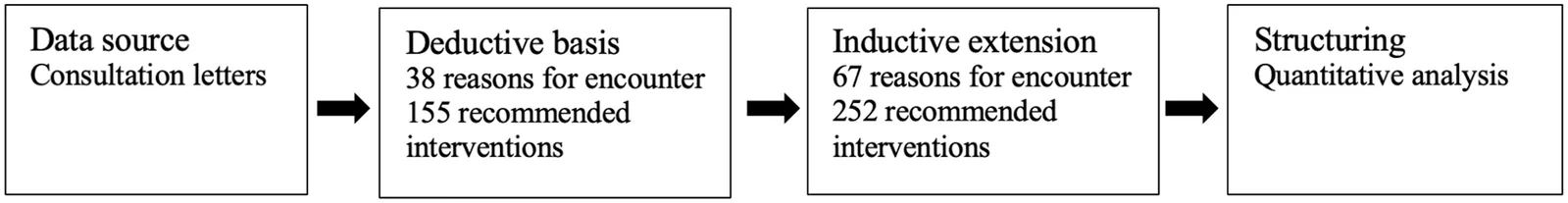
Flow analysis.

### 2.5 Subgroup analysis

Subgroup analyses by stage of diagnosis, treatment intent, and primary cancer diagnosis were performed to account for clinically relevant heterogeneity in patient populations and to assess whether counseling content and recommendations varied across these clinically distinct groups. To determine which measures were used for specific symptoms, we conducted a subgroup analysis of the recommended interventions for some of the most common RfE. This allows the spectrum of CIH interventions available for specific symptoms to be described in more detail and, where appropriate, to be addressed in subsequent studies or training programs.

### 2.6 Ethical considerations

The study was registered and approved by the ethics committees of the University of Tuebingen, Germany (No. 658/2019BO1; Trial Registration Number, DRKS00021779). Comprehensive informed consent had been obtained for all data collected in the study.

## 3 Results

### 3.1 Study population

A total of 685 patients were initially enrolled in the intervention group of the CCC-Integrativ study. Of these, 82 participants did not complete the end-of-intervention questionnaire (T2) administered after the three scheduled consultations, representing a dropout rate of 12% (51). In accordance with the analytic strategy of the primary study evaluation, the consultation letters from these participants were excluded from the present analysis. The final study cohort comprised *n* = 603 patients with available consultation letters. Of these, *n* = 116 letters were not included in the analysis because they had either not been written or contained neither new RfE nor new CIH interventions, resulting in a final analysis of *n* = 1,693 documented consultations.

### 3.2 Sociodemographic and clinical characteristics

The sociodemographic and clinical characteristics of the analyzed cohort are summarized in Table 1. The majority of participants were female (*n* = 463, 76.8%). Patient age ranged from 18 to 88 years, with a mean age of 55 years (SD ± 11.4). A university degree or equivalent qualification was held by 249 participants (41.6%), and 378 participants (62.7%) were employed. Most patients lived in a stable partnership (*n* = 468, 79.6%). The mean body mass index (BMI) was 24.5 kg/m2 (SD ± 4.77).

**Table 1:** Sociodemographic characteristics (sample sizes vary across variables due to missing data).

Total *n* = 603	*n* (%)	N*
Socio-demographics		
Gender		603
Male	140 (23.2%)	
Female	463 (76.8%)	
Mean age (SD) in years	55.4 (SD 11.4)	603
Education		599
Secondary school	171 (28.5%)	
High school	109 (18.2%)	
University/collegue degree	249 (41.6%)	
None/basic secondary school (8 years)	70 (11.7%)	
Employment status		598
Full-time	206 (34.4%)	
Part-time	172 (28.8%)	
Not working	45 (7.53%)	
Retired	175 (29.3%)	
Marital status		588
Single	120 (20.4%)	
In a relationship/married	468 (79.6%)	
Mean BMI (SD) in kg/m2	24.5 (SD 4.77)	590
Clinical data		
Stage of diagnosis		603
Initial diagnosis	365 (60.5%)	
Progression	171 (28.4%)	
Relapse	67 (11.1%)	
Treatment intent		603
Curative	320 (53.1%)	
Palliative	202 (33.5%)	
Unclear	81 (13.4%)	
Chronic condition:		603
Yes	368 (61.0%)	
No	235 (39.0%)	
Primary cancer diagnosis		603
Breast Cancer	276 (45.8%)	
Gastrointestinal	105 (17.4%)	
Gynecological	50 (8.29%)	
Respiratory/Thoracic	36 (5.97%)	
Other	136 (22.6%)	
Metastases:		556
No	329 (59.2%)	
Yes	227 (40.8%)	

Regarding disease characteristics, 365 patients (60.5%) were enrolled following an initial cancer diagnosis, 171 (28.4%) with disease progression, and 67 (11.1%) with cancer recurrence. The treatment intent was curative in 320 patients (53.1%). A total of 368 patients (61.0%) had a chronic condition, which was defined as the coexistence of at least one chronic disease in addition to cancer. A broad spectrum of cancer entities was covered, with breast cancer being the most common diagnosis (*n* = 276 patients, 45.8%), followed by gastrointestinal malignancies (*n* = 105 patients, 17.4%). Metastatic disease was already present in 40.8% of the enrolled patients (Table 1. Note: sample sizes vary across variables due to missing data).

### 3.3 Reasons for encounter

Across the 603 patients, a total of 4,241 RfE were identified, corresponding to an average of approximately seven reasons per patient. The most frequently reported specific consultation topics included general weakness and tiredness (cancer-related fatigue; *n* = 336, 56%), general questions regarding complementary medicine (44%), chemotherapy-induced peripheral neuropathy (CIPN; 40%), sleep disturbances (37%), and general pain (36%) (Table 2).

**Table 2:** Specific reasons for encounter.

Specific reasons for encounter	ICPC-3	*n* = 603	%
Cancer-related fatigue	AS04	336	56%
General questions about CIH	AS99	264	44%
Chemotherapy induced peripheral neuropathy	NS04	244	40%
Sleep disturbances	PS06	226	37%
Pain	AS01	215	36%
Skin reactions	SS08	199	33%
Mental stress	ZC99	195	32%
Nausea	DS09	193	32%
Mucositis and xerostomia	DS20	184	31%
Anxiety/restlessness	PS01	153	25%
General digestive complaints	DS07	149	25%
Dysgeusia	NS08	138	23%
General mucosal problems	SSXX	127	21%
Diarrhea	DS11	99	16%
Loss of appetite	TS03	96	16%
Constipation	DS12	95	16%
Nail changes	SS09	78	13%
Menopausal symptoms (hot flashes)	GS13	75	12%
Lymphedema	AS09	64	11%
Cold/heat sensitivity	AS02	63	10%
Hand-foot syndrome	SS99	41	7%

CIH, Complementary and Integrative Healthcare; ICPC-3, International Classification of Primary Care – 3. Multiple answers are allowed for RfE.

### 3.4 Lifestyle modifications and specific CIH interventions

Lifestyle modifications were addressed in the majority of consultations. Nutritional counseling was provided to 570 patients (95%), physical activity was discussed with 558 patients (93%), and a total of 487 (81%) patients were offered guidance on stress management or relaxation strategies.

Across all consultations, a total of 9,045 CIH interventions were recommended, representing an average of approximately 15 recommendations per patient. These interventions were categorized into three main groups: non-pharmacological interventions (NPI), naturopathic nursing interventions (NNI), and medicines and teas (MT). Of all recommendations, 4,107 (45%) belonged to the NPI category, 3,740 (41%) were NNI, and 1,198 (13%) were MT.

NPI were recommended to 588 patients (98%). Acupressure was the most frequent intervention, recommended to 475 patients (79%). Other common recommendations included the provision of informational materials or referrals to additional supportive resources such as subsequent counseling sessions at the four CCCs (73%), individualized nutritional counseling (61%), individualized exercise counseling (53%), mindfulness-based movement therapies such as Yoga, Tai Chi, or Qigong (44%), and structured relaxation techniques (29%).

Given that 583 patients (97%) received NNI, these interventions also constituted a substantial proportion of the recommendations. The most frequently suggested measures included skin care interventions (57%), therapeutic rubs (55%), oil or ointment compresses (51%), and partial or full therapeutic baths (51%).

Recommendations involving MT were slightly less frequent than those for NPI and NNI, applying to 475 patients (79%). Herbal teas were the most commonly recommended preparations (59%), followed by phytotherapeutics (55%) and anthroposophic medicinal products (10%). Figure 2 shows the lifestyle modifications and the most frequent recommendations according to the three groups NPI, NNI and MT (Figure 2).

**Figure 2:**
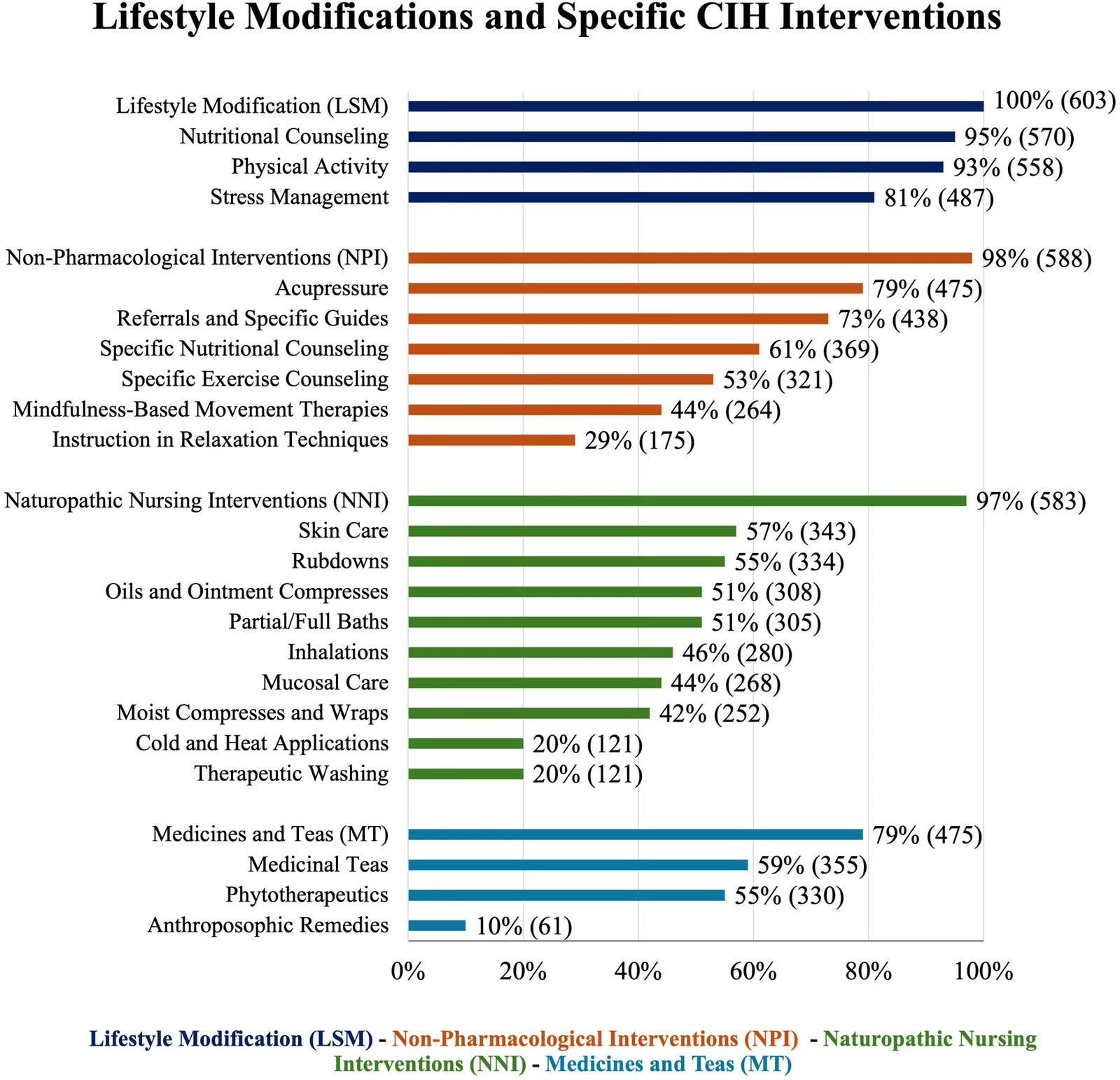
Lifestyle modifications and specific CIH interventions in the total study cohort.

### 3.5 General subgroup analysis

#### 3.5.1 Stage of diagnosis

Patients were initially categorized according to their stage of diagnosis, namely initial diagnosis (*n* = 365; 61%), progression (*n* = 171; 28%), and relapse (*n* = 67; 11%). In terms of RfE, some differences were observed between the three groups. Chemotherapy-induced peripheral neuropathy (CIPN) and skin reactions were documented more frequently in the initial diagnosis group, whereas cancer-related fatigue, general questions, and pain were documented most frequently in the progression group. Sleep disturbances were most frequent in the relapse group (Table 3). In terms of CIH interventions, the number of patients receiving lifestyle modifications was comparable across all three groups, whereas the specific CIH interventions differed. In the field of NPI, the progression group had particularly high numbers of recommendations for referrals and specific guides, and specific physical activity and nutritional counseling. The progression group also had higher numbers of recommendations for mindfulness-based movement therapies. In the field of NNI, rubs and inhalations were recommended more frequently in the progression group, whereas partial and full baths were recommended more frequently in the relapse group. In the field of MT, the relapse group received the lowest number of recommendations (Table 4; Figure 3).

**Table 3:** Specific reasons for encounter by stage of diagnosis.

Reason for encounter	Total	%	Initial Diagnosis	%	Progression	%	Relapse	%
	*n* = 603	100%	*n* = 365	61%	*n* = 171	28%	*n* = 67	11%
Cancer-related fatigue	336	56%	193	53%	110	64%	33	49%
General questions about CIH	264	44%	154	42%	85	50%	25	37%
CIPN	244	40%	163	45%	55	32%	26	39%
Sleep disturbances	226	37%	130	36%	67	39%	29	43%
Pain	215	36%	114	31%	78	46%	23	34%
Skin reactions	199	33%	140	38%	40	23%	19	28%
Mental stress	195	32%	121	33%	56	33%	18	27%
Nausea	193	32%	118	32%	59	35%	16	24%
Mucositis and xerostomia	184	31%	117	32%	52	30%	15	22%
Anxiety/restlessness	153	25%	93	25%	48	28%	12	18%
General digestive complaints	149	25%	88	24%	47	27%	14	21%
Dysgeusia	138	23%	98	27%	29	17%	11	16%
General mucosal problems	127	21%	79	22%	35	20%	13	19%
Diarrhea	99	16%	57	16%	33	19%	9	13%
Loss of appetite	96	16%	57	16%	31	18%	8	12%
Constipation	95	16%	53	15%	27	16%	15	22%
Nail changes	78	13%	60	16%	12	7%	6	9%
Menopausal symptoms (hot flashes)	75	12%	58	16%	14	8%	3	4%
Lymphedema	64	11%	38	10%	20	12%	6	9%
Cold/heat sensitivity	63	10%	42	12%	14	8%	7	10%
Hand-foot syndrome	41	7%	16	4%	21	12%	4	6%

CIH, Complementary and Integrative Healthcare; CIPN, Chemotherapy-Induced Peripheral Neuropathy, Multiple answers are allowed for RfE.

**Table 4:** Lifestyle modifications and specific CIH interventions by stage of diagnosis.

	Total	%	Initial diagnosis	%	Progression	%	Relapse	%
Intervention	*n* = 603	100%	*n* = 365	100%	*n* = 171	28%	*n* = 67	11%
*Lifestyle modification (Total)*	603	100%	365	100%	171	100%	67	100%
Nutritional counseling	570	95%	348	95%	160	94%	62	93%
Physical activity	558	93%	343	94%	156	91%	59	88%
Relaxation strategies	487	81%	302	83%	132	77%	53	79%
*Non-pharmacological interventions (NPI)*	588	98%	354	97%	169	99%	65	97%
Acupressure	475	79%	285	78%	137	80%	53	79%
Referrals and specific guides	438	73%	261	72%	130	76%	47	70%
Specific nutritional counseling	369	61%	216	59%	115	67%	38	57%
Specific exercise counseling	321	53%	182	50%	106	62%	33	49%
Mindfulness-based movement therapies	264	44%	160	44%	70	41%	34	51%
Instruction in relaxation techniques	175	29%	109	30%	48	28%	18	27%
*Naturopathic nursing interventions (NNI)*	582	97%	348	95%	169	99%	65	97%
Skin care	343	57%	216	59%	90	53%	37	55%
Rubdowns	333	55%	182	50%	114	67%	37	55%
Partial/full baths	304	50%	181	50%	84	49%	39	58%
Oils and ointment compresses	308	51%	185	51%	90	53%	33	49%
Inhalations	280	46%	160	44%	91	53%	29	43%
Mucosal care	229	38%	139	38%	68	40%	22	33%
Moist compresses and wraps	241	40%	144	39%	69	40%	28	42%
Therapeutic washing	121	20%	75	21%	36	21%	10	15%
Cold and heat applications	120	20%	75	21%	33	19%	12	18%
*Medicines and teas (MT)*	475	79%	291	80%	137	80%	47	70%
Medicinal teas	355	59%	217	59%	108	63%	30	45%
Phytotherapeutics	329	55%	205	56%	89	52%	35	52%
Anthroposophic remedies	61	10%	40	11%	17	10%	4	6%

CIH, Complementary and Integrative Health Care. Multiple answers are allowed for Interventions.

**Figure 3:**
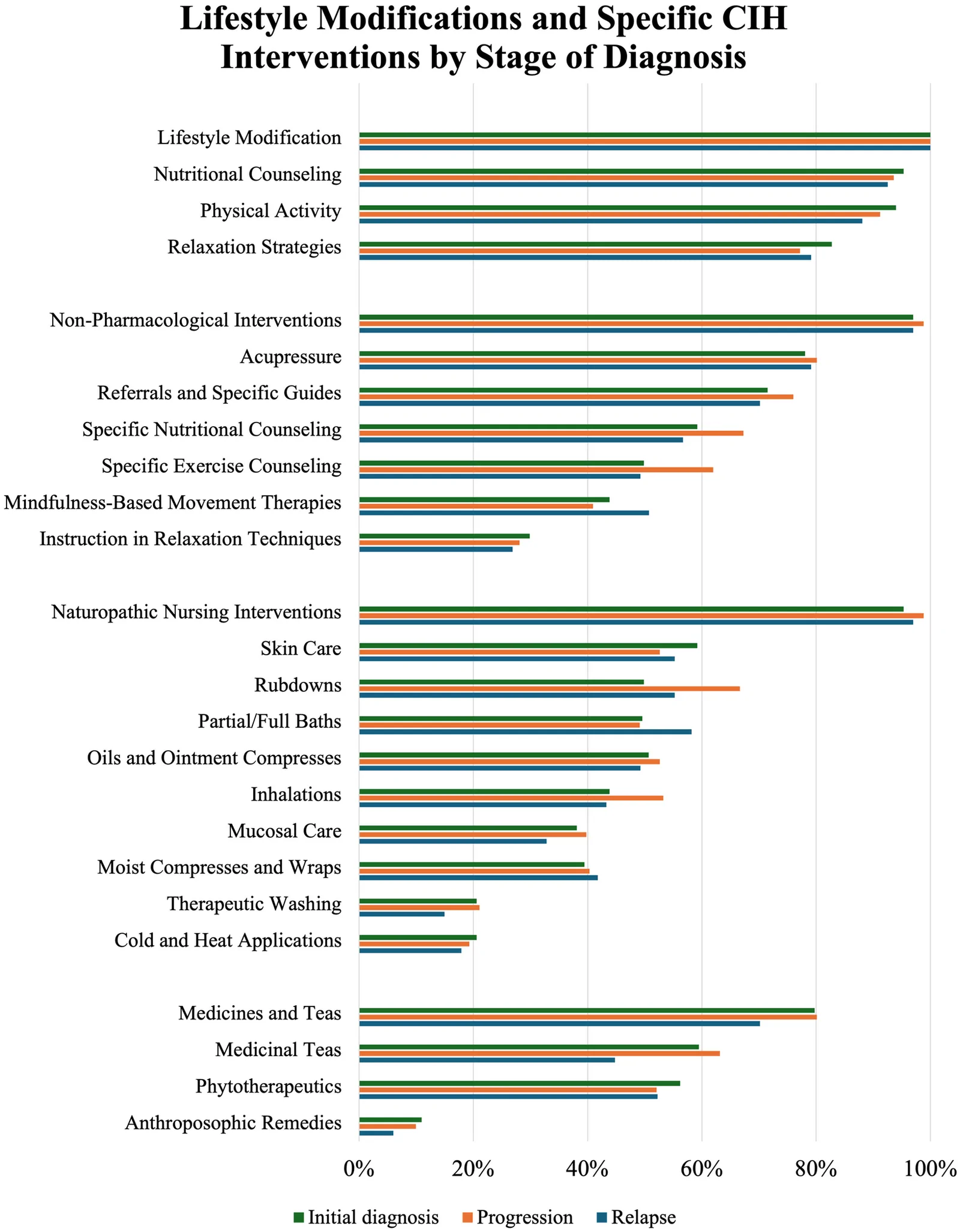
Lifestyle modifications and specific CIH interventions by stage of diagnosis.

#### 3.5.2 Treatment intent

Patients were categorized according to treatment intent: curative (*n* = 320; 53%), palliative (*n* = 202; 33%), or unclear (*n* = 81; 13%). The curative group reported higher incidences of chemotherapy-induced peripheral neuropathy and skin reactions. In contrast, palliative patients exhibited elevated levels of cancer-related fatigue, pain, and mental stress as RfE (Supplementary 3). In the case of lifestyle modifications and CIH interventions, a greater number of relaxation strategies were discussed with the curative group. However, the palliative group more frequently received referrals and specific guides, as well as specific physical activity (PA) and nutritional counseling, rubs and inhalations, and medicinal teas and phytotherapeutics (Supplementary 4).

#### 3.5.3 Cancer diagnosis

The most frequent cancer diagnoses were as follows: breast cancer (*n* = 276; 46%), gastrointestinal cancers (*n* = 105; 17%), gynecological cancers (*n* = 50; 8%), respiratory/thoracic cancers (*n* = 36; 6%), and others (*n* = 136; 23%). Differences were also observed in the RfE. Cancer-related fatigue, sleep disturbances, and skin reactions were reported most frequently by patients with breast cancer. However, patients with gastrointestinal cancers had the highest number of cases of CIPN, and patients with gynecological cancer reported more pain (Supplementary 5).

Lifestyle modifications were discussed with patients with gastrointestinal cancers less frequently than with other groups, but they did receive more specific nutritional counseling. However, the highest number of referrals and specific guides was received by breast cancer patients (Supplementary 6).

### 3.6 Symptom-specific subgroup analyses

To explore symptom-specific recommendations, subgroup analyses were conducted for five frequently reported and clinically relevant RfE in supportive oncology: fatigue, chemotherapy-induced peripheral neuropathy (CIPN), sleep disturbances, pain, and nausea. For some patients, multiple interventions from the same category were recommended. The percentages indicate the proportion of patients who received a specific intervention for the corresponding symptom.

#### 3.6.1 General weakness and tiredness

General weakness and tiredness (fatigue; ICPC-3: AS04) were reported as RfE by 336 patients (56%) across the three counseling sessions. The coding framework for analyzing the consultation letters encompassed fatigue, tiredness, exhaustion, and the need for activation. A total of 62 distinct interventions were identified for this symptom cluster. Because general lifestyle modifications (such as nutrition, physical activity, and stress regulation) had already been provided to nearly all patients, this analysis includes only specific or advanced recommendations.

Within NPI, individualized exercise counseling was the most frequently recommended intervention (*n* = 157; 47%), followed by acupressure for fatigue (*n* = 73; 22%) and mindfulness-based movement therapies (*n* = 43; 13%), including Yoga, Tai Chi, and Qigong. Additionally, Yoga alone was explicitly recommended to a smaller group (*n* = 16; 5%).

Among NNI, aromatherapy applications using rosemary (Rosmarinus officinalis) – either alone or combined with citrus oils – were most frequently used (*n* = 108; 32%). Additional common recommendations included moist-hot yarrow liver compresses (*n* = 83; 25%), morning foot baths with rosemary (*n* = 77; 23%), therapeutic washes with rosemary (*n* = 67; 20%), Kneipp hydrotherapy applications (*n* = 44; 13%), lemon-based foot applications (*n* = 27; 8%), and related topical or aromatherapeutic interventions.

MT played a comparatively minor role. Here, the most frequently recommended options were green tea (Camellia sinensis; *n* = 42; 13%), mistletoe preparations (Viscum album; *n* = 36; 11%), ginger tea (Zingiber officinale; *n* = 19; 6%), and ginseng (Panax ginseng; *n* = 18; 5%) (Figure 4).

**Figure 4:**
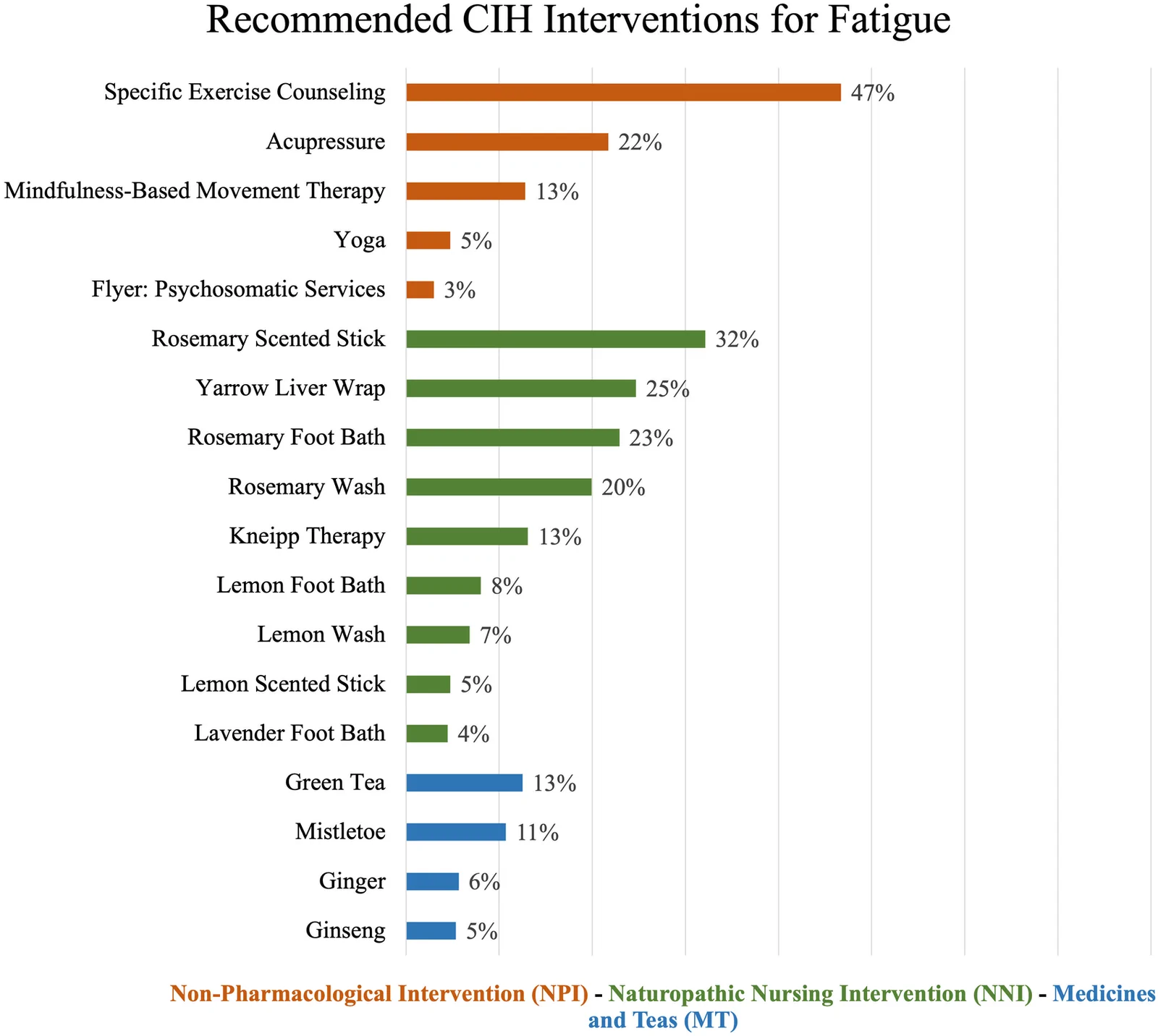
Specific CIH interventions for fatigue.

#### 3.6.2 Chemotherapy-induced peripheral neuropathy

Chemotherapy-induced peripheral neuropathy (CIPN; ICPC-3: NS04) was addressed in 244 patients (40%). The most commonly recommended NPI was acupressure for CIPN (*n* = 163; 67%). Further frequently applied interventions included structured informational materials (*n* = 75; 31%), balance and coordination exercises (*n* = 73; 30%), seed-based sensory stimulation baths (*n* = 61; 25%), and self-massage using spiked balls (*n* = 60; 25%). Additional recommendations comprised mindfulness-based movement therapies (*n* = 43; 18%), exercise counseling (*n* = 42; 17%), sensory training exercises (*n* = 38; 16%), as well as Qigong (*n* = 16; 7%) and Tai Chi (*n* = 12; 5%).

Among NNI, a sugar-oil peeling (using edible oil and sugar) was the predominant intervention (*n* = 146; 60%), followed by topical applications using Aconite pain relief oil (comprising Lavandulae aetheroleum, D-camphor, Quarz Dil. D9 oleos, and Aconitum napellus e tubere ferm 33c Dil. D9 oleos; *n* = 64; 26%). No MT were documented for CIPN (Figure 5).

**Figure 5:**
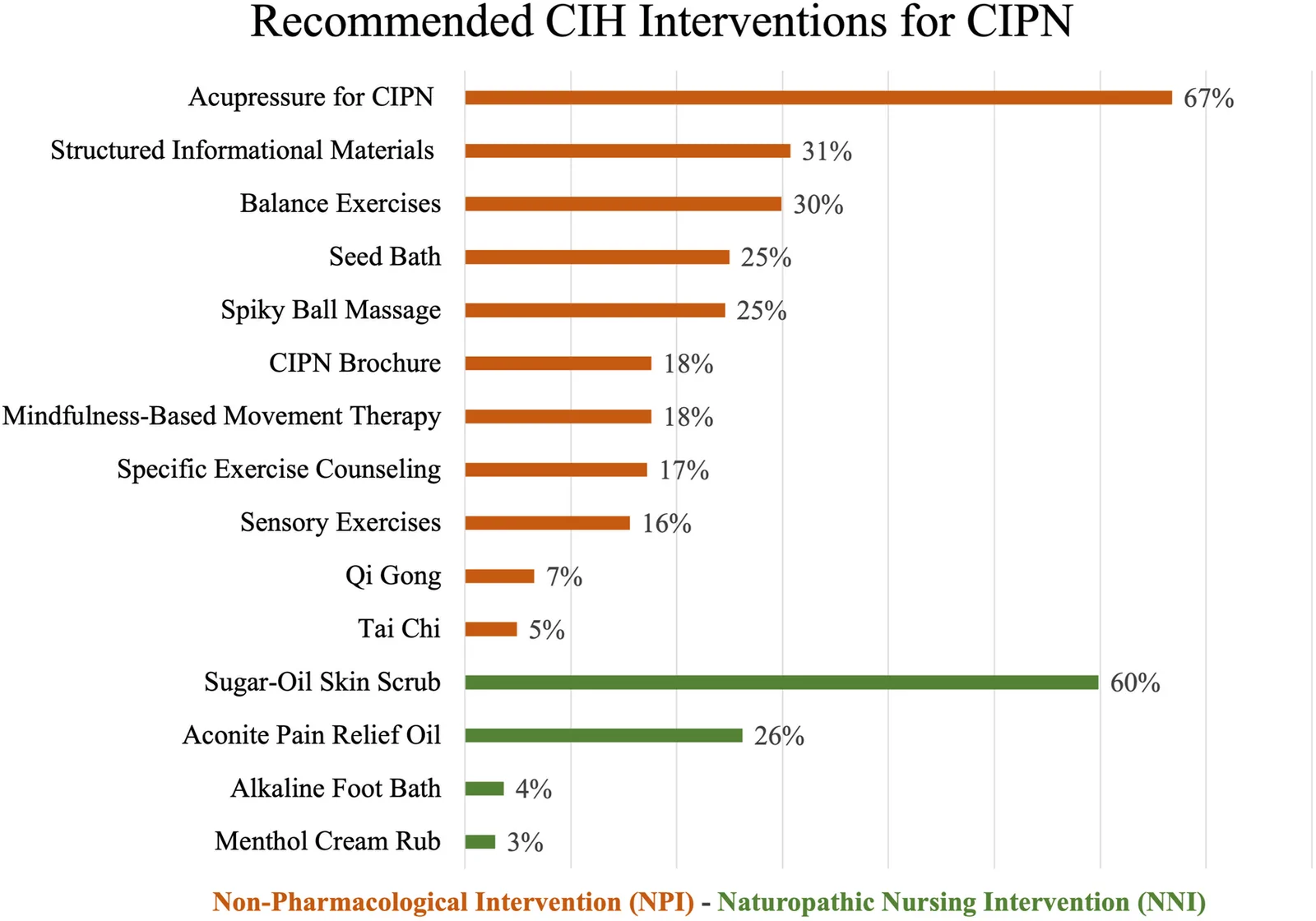
Specific CIH interventions for CIPN.

#### 3.6.3 Sleep disturbances

Sleep disturbances (ICPC-3: PS06) were reported in 226 patients (37%). Acupressure was the most frequently recommended NPI (*n* = 83; 37%), followed by breathing techniques (*n* = 60; 27%). Less frequently recommended interventions included mindfulness-based movement therapies (*n* = 15; 7%) such as Yoga, Tai Chi, and Qigong, mindfulness-based stress reduction (MBSR; *n* = 13; 6%), and referrals to psychosocial support services (*n* = 10; 4%).

Within NNI, heart compresses using Aurum/Lavandula comp. (comprising Lavandulae aetheroleum, Aetheroleum extractum e floribus recentibus Rosae damascenae et centifoliae, and Aurum metallicum praeparatum Dil. D4) were most commonly recommended (*n* = 106; 47%). Lavender-based applications were also frequently utilized, including foot baths (*n* = 65; 29%), aromatherapy sticks (*n* = 26; 12%), and foot rubs (*n* = 11; 5%). Moist-hot yarrow liver compresses were applied in a smaller subgroup of cases (*n* = 10; 4%).

Among MT, herbal sleep teas (comprising Valeriana officinalis, Humulus lupulus, Melissa officinalis, and Passiflora incarnata) were most frequently recommended (*n* = 62; 27%), followed by valerian (Valeriana officinalis) (*n* = 20; 9%), Cardiodoron/Aurum comp. (comprising Onopordum acanthium, Primula veris, Hyoscyamus niger, Arnica montana ex planta tota D10, Aurum metallicum praeparatum D10, and Formica rufa D10; *n* = 14; 6%), and Lasea capsules (ingredients: Lavandula angustifolia Mill., aetheroleum; *n* = 10; 4%) (Figure 6).

**Figure 6:**
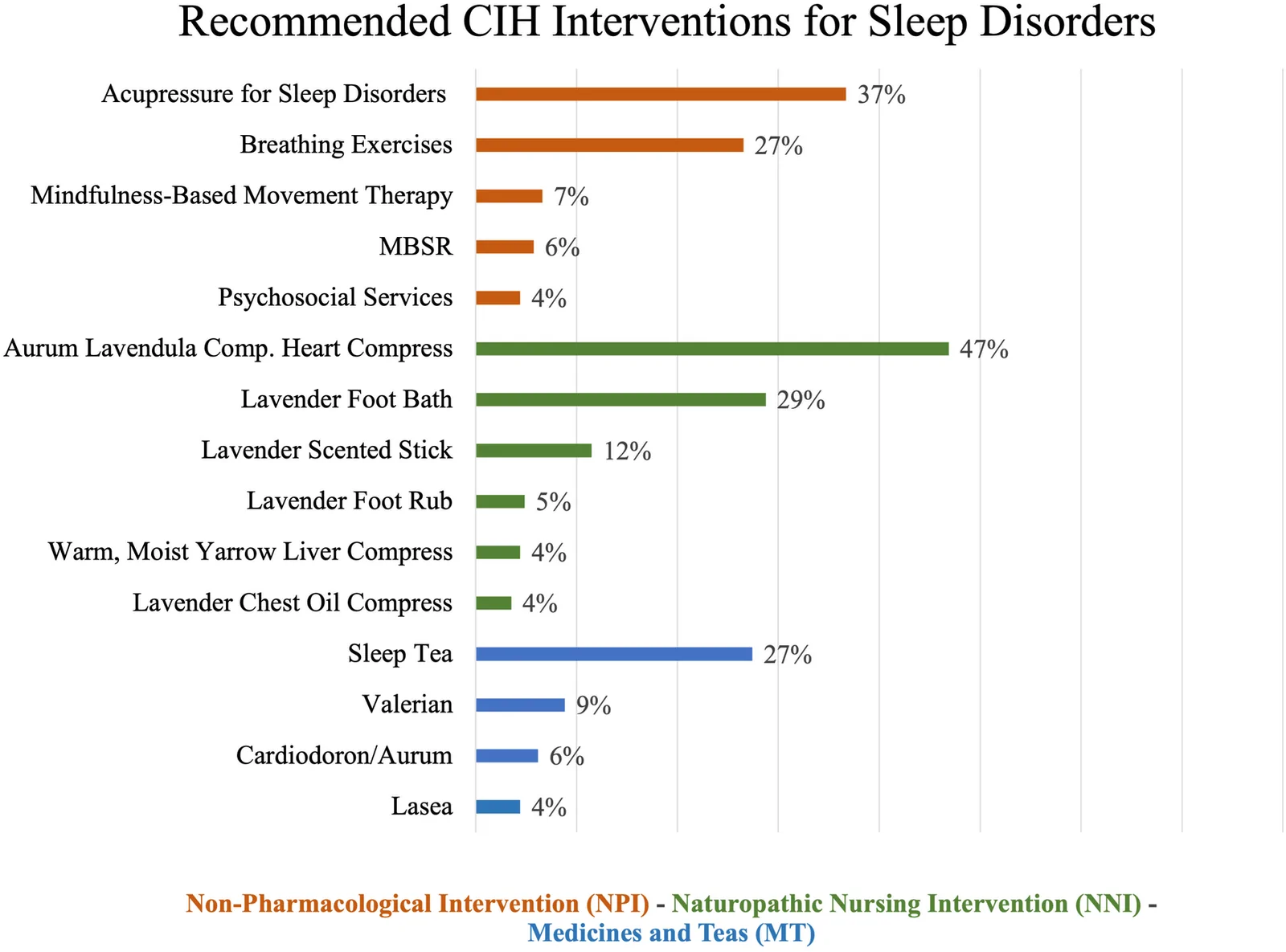
Specific CIH interventions for for sleep disturbances.

#### 3.6.4 General pain

General pain (ICPC-3: AS01) was addressed in 215 patients (36%). Acupressure represented the most frequently recommended NPI (*n* = 64; 30%). Additional interventions included individualized exercise counseling (*n* = 14; 7%), Yoga (*n* = 7; 3%), referral to specialized pain services (*n* = 6; 3%), and referral for acupuncture (*n* = 6; 3%). NNI were predominantly based on topical applications. Embrocations using Aconite pain relief oil (comprising Lavandulae aetheroleum, D-Camphor, Quarz Dil. D9 oleos, Aconitum napellus e tubere ferm 33c Dil. D9 oleos.) were most common (*n* = 121; 56%), followed by Solum oil applications (comprising Solum uliginosum, Aesculus hippocastanum e semine LA 20%, Equisetum arvense ex herba LA 20%, Lavandulae aetheroleum; *n* = 33; 15%), thermotherapy (*n* = 24; 11%), cabbage leaf compresses (*n* = 14; 7%), quark compresses (*n* = 13; 6%), and Solum oil compresses (*n* = 10; 5%). No MT were reported for pain (Figure 7).

**Figure 7:**
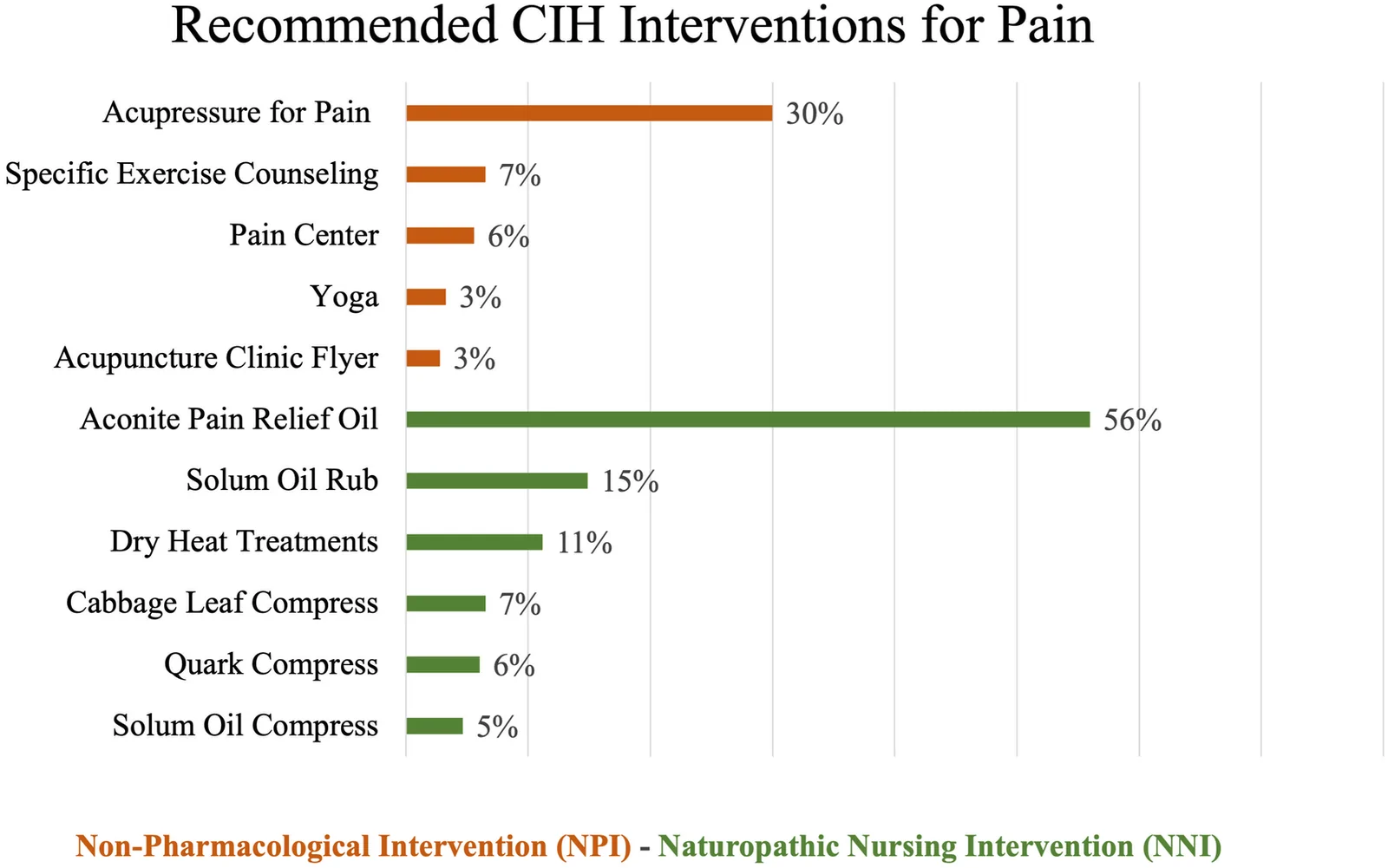
Specific CIH interventions for pain.

#### 3.6.5 Nausea

Nausea (ICPC-3: DS09) was reported in 193 patients (32%). Acupressure was the most frequently recommended NPI (*n* = 149; 77%), followed by acupressure using Sea-Bands (*n* = 43; 22%) and breathing exercises (*n* = 5; 3%). Among NNI, aromatherapy interventions were predominant, particularly lemon-based essential oil applications (*n* = 76; 39%), followed by bergamot (*n* = 34; 18%) and peppermint (*n* = 9; 5%). Additional measures included abdominal oil compresses using fennel (*n* = 8; 4%) and orange-based aromatherapy (*n* = 5; 3%). Within MT, ginger tea was most frequently recommended (*n* = 76; 39%), followed by Nux vomica comp. (comprising Gentiana lutea ethanol. decoctum Dil. D1, Nux moschata Dil. D4, Nux vomica Dil. D4; *n* = 16; 8%), bitter herbal preparations such as Amara drops (comprising Artemisia absinthium, Centaurium erythraea, Cichorium intybus, Gentiana lutea, Imperatoria ostruthium, Juniperus communis, Salvia officinalis, Achillea millefolium; *n* = 9; 5%), and bitter tea (comprising Artemisia absinthium, Centaurium erythraea, Gentiana lutea, Achillea millefolium; *n* = 9; 5%) (Figure 8).

**Figure 8:**
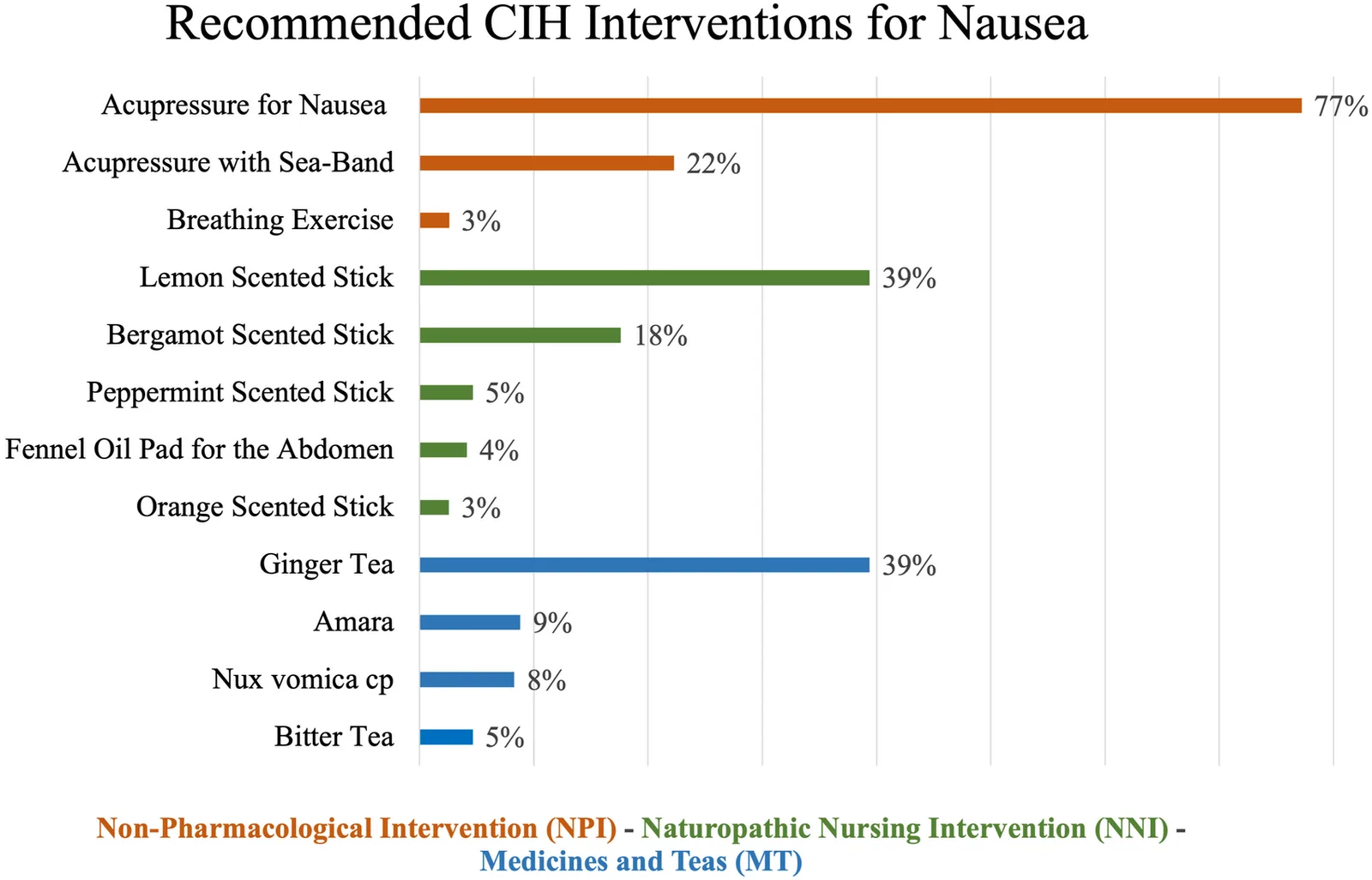
Specific CIH interventions for nausea.

## 4 Discussion

In the context of the CCC-Integrativ study, the present study investigated the specific RfE recorded for oncology patients in outpatient counseling across four CCCs in Baden-Württemberg, Germany, as well as the recommended CIH interventions. The RfE covered a wide range of common symptoms experienced by cancer patients, including fatigue, CIPN, sleep disturbances, pain, skin reactions, mental stress, nausea, mucositis, anxiety, and digestive complaints. Lifestyle modifications were discussed with nearly all patients. On average, 14 specific recommendations were provided per patient in the areas of NPI, NNI, and MT tailored to their individual needs.

The cohort was predominantly female, relatively young, and well-educated, with a high proportion of breast cancer cases. This distribution corresponds to known usage patterns of complementary therapies (67, 68).

Within the framework of the CCC-Integrativ counseling service, one hour was allocated for the initial interprofessional consultation, followed by 30 min for each of the two follow-up appointments. A process evaluation showed that this approach was highly valued by patients, as evidenced by accompanying interviews conducted within the project (64). Almost all patients received advice on lifestyle modifications, such as nutrition (95%), physical activity (93%), and stress management (81%). These three pillars are central to prevention, treatment, and rehabilitation in oncology as well as in other fields of medicine and healthcare, especially in primary care. They provide a strong and growing body of evidence demonstrating positive effects on treatment outcomes (12), quality of life, a variety of symptoms (14, 24–27), and survival in primary prevention (16) as well as across all stages of disease (17, 18). Furthermore, data suggest that the combination of different approaches in particular, such as nutrition and exercise, can significantly reduce mortality risk in cancer patients (69), highlighting the importance of providing thorough information on lifestyle modifications. In addition to basic advice on lifestyle modifications, around half of the patients received specific nutritional counseling (61%), tailored exercise recommendations or referrals (53%), and recommendations for mindfulness-based movement therapies (44%). This demonstrates the extent to which outpatient CIH was able to provide individualized, in-depth guidance tailored to patients’ specific needs in addition to general advice on lifestyle modifications. In contrast, studies indicate that regular oncological consultations often lack sufficient time for individual lifestyle modifications (70) and do not sufficiently address the informational and emotional needs of the patients (71).

Regarding CIH interventions, 79% of patients received recommendations for acupressure for various tumor-associated symptoms, making it the most frequently recommended NPI. This largely corresponds with the results from numerous systematic reviews demonstrating positive evidence for acupressure or acupuncture in managing various symptoms, as reflected in national (8) and international (72) guideline recommendations. Similar, mindfulness-based movement therapies including Yoga, Tai Chi, and Qigong were recommended to 44% of patients, which aligns with their importance in German and international guidelines (8, 11, 73). Furthermore, 73% of patients received referrals to specialized outpatient clinics or specific educational resources. Patients were referred to a range of counseling services at the University Hospital, including psychosocial and psycho-oncological counseling, the acupuncture clinic, the nursing consultation service, the pain clinic, and specialized exercise and nutrition counseling. They were further referred to the Cancer Information Service, the patient version of the S3-Guideline Complementary Medicine in the Treatment of Oncological Patients, and topic-specific educational brochures addressing symptoms such as chemotherapy-induced peripheral neuropathy (CIPN) and hand-foot syndrome, among others. In addition, patients received information and links to help them find qualified physicians and therapists offering CIH interventions as relaxation techniques and specific naturopathic nursing interventions, as well as apps for meditation and relaxation practices. Overall, these findings indicate a close integration of CIH counseling within conventional and complementary care pathways and local care networks. They also highlight the important navigational role of CIH counseling, which may serve a screening purpose for unmet patient needs and support patients in navigating a broad range of services in demanding clinical situations.

Nearly all patients (97%) received recommendations for NNI. While large clinical trials are lacking for many of these interventions, a substantial body of experiential knowledge exists, commonly conveyed through textbooks and continuing professional education. Furthermore, systematically developed expert consensus is available for several of these interventions, some of which have already been described in the literature (34, 36, 37); however, guideline recommendations for NNI remain scarce. This is in accordance with Sackett's model of evidence-based medicine, in which the evidence base for NNI rests less on the first pillar – best available external research evidence – and more strongly on the second and third pillars: clinical expertise accumulated through practice and professional training, and patient values and preferences (74). This approach to selecting CIH interventions was reflected in the high acceptance and appreciation of these recommendations, as shown in previous qualitative studies on the CCC-Integrativ counseling program (63, 75). The wide range, high frequency, and interprofessional nature of these interventions represented a distinctive feature of CCC-Integrativ consultations. This highlights the close collaboration between medical and nursing experts, which was also highly valued by patients (75).

MT recommendations played a minor role, accounting for only 13% of all recommendations. This is expected, as the primary goal of the study was not to administer pharmacological treatments, but to enhance patient activation primarily through non-pharmacological interventions (61). Indeed, the most frequently recommended phytotherapeutics are already included in oncological guidelines, such as Zingiber officinale, Viscum album, and Panax ginseng (8) or are classified as well-established or traditionally used substances, including Plantago ovata (76), Lavandula angustifolia (77), and Valeriana officinalis (78).

### 4.1 General subgroup analysis

In the subgroup analysis according to stage of diagnosis, CIPN and skin reactions were more frequent in the initial diagnosis group. The highest symptom burden (fatigue and pain) and the highest number of NPI recommendations (referrals and mindfulness-based interventions) were documented at progression. Sleep disturbances were more frequent at relapse. This is consistent with reports linking longer disease duration to a stronger association between fatigue and interest in complementary and integrative medicine (79, 80). The elevated rate of sleep disturbances in the relapse group aligns with evidence that fear of recurrence and cancer-related distress are key drivers of poor sleep in long-term survivors (81). The more intensive NPI recommendations in the progression group mirror the general principle that integrative approaches broaden as illness advances (82).

### 4.2 Symptom-specific subgroup analyses

Frequent RfE in our study included cancer-related fatigue (CRF; 56%), general questions regarding complementary medicine (44%), CIPN (40%), sleep disturbances (37%), pain (36%), and nausea (32%). Current studies report a prevalence of CRF ranging from 49% (83) to 52% (84) or even higher (85). CIPN is one of the most prevalent and disabling side effects of antineoplastic therapies, with a prevalence of approximately 48% (86, 87), and high long-term persistence (88). The prevalence of sleep disorders among cancer patients reported in the literature ranges from 57% (89) to 60.7% (90), which is approximately 20 percentage points higher than in our cohort. In recent studies, the prevalence of pain is estimated at 44.5% (91) and that of nausea at 48% (85), which is around 12 to 16 percentage points higher than observed in our cohort. These data indicate that the prevalence of these symptoms within our study was mostly within or below published ranges, suggesting that our CCC-Integrativ consultation provided a realistic representation of the situation of outpatient cancer patients.

For cancer related fatigue, studies indicate that it is associated with additional risks, including treatment interruptions and significant impacts on social functioning, self-management, and quality of life (92). The recommended NPI and MT interventions for cancer related fatigue in our study were largely consistent with guideline recommendations and additional study data demonstrating the effectiveness of physical activity (13), mindfulness-based movement therapies (8, 25–27), acupressure (8, 93, 94), mistletoe therapy (95), and ginseng (8). The rate for mindfulness-based movement therapies appears low (13% for mindfulness-based movement therapies in general, plus 5% specifically for Yoga) compared to the strong recommendations in the guidelines mentioned above. However, our data show also that the overall rate of patients receiving mindfulness-based movement therapies was 44%. This gap may be explained by the fact that these therapies had probably already been recommended to the same patients for other symptoms and were therefore not included in the RfE for the specific symptom examined. As patients often presented with several coexisting symptoms rather than sleep disorders, fatigue, or CIPN alone, the counseling team and the patient reached an informed consensus on which intervention – NPI, NNI, or MT – to recommend for which symptom, and, where several options were available for the same symptom, on which of these to prefer, typically prioritizing those causing the greatest burden. In this process, NPI and NNI were generally given preference over MT, reflecting both the more stringent safety criteria applied to pharmacological interventions during catalog development and the program's focus on patient activation. The NNI applied had limited high-level evidence but demonstrated favorable profiles in terms of patient self-efficacy, interaction potential, and cost-effectiveness (34, 96, 97).

Recommendations for CIPN focused on functional and sensory training approaches, including mindfulness-based movement therapies and acupressure. These were largely guideline-concordant (8) or supported by recent studies demonstrating significant improvements in self-reported CIPN under combined interventions, such as Qigong and acupressure (98). Recommended NPI also aimed to stimulate tactile sensory input, for example through sugar-oil peelings and embrocations using Aconite pain relief oil (comprising Lavandulae aetheroleum, D-camphor, Quarz Dil. D9 oleos., Aconitum napellus ex tubere ferm 33c Dil. D9 oleos.).

Concerning sleep disorders, the NPI in our study covered acupuncture, breathing exercises, mindfulness-based movement therapies, and mindfulness-based stress reduction (MBSR). This is consistent with recommendations in the German S3 guideline, “Complementary Medicine in the Treatment of Oncological Patients,” which notes positive evidence for Tai Chi, Qigong, MBSR, and acupuncture (8). Additional data also indicate that acupressure is effective for sleep disorders (99–101). None of the suggested NNI and MT carried guideline recommendations; however, for MT recommendations and the clinical use of lavender as an NNI, there is some supportive evidence from smaller studies (102–105).

### 4.3 International comparison of reasons for encounter and recommended interventions

One of the most widely cited references, an analysis of IO consultations at MD Anderson Cancer Center (USA), examined 2,367 patients and found that the most common RfE were seeking a general integrative approach or guidance on herbs/supplements and nutrition, while specific symptoms accounted for only up to 13% of concerns (106). These results differ fundamentally from our analysis; however, it should be noted that this study used the MYCaW instrument, a prospective, patient-reported tool in which patients themselves nominate and prioritize their one or two main concerns, whereas our study analyzed the consultation letters, capturing the full range of RfE addressed during the encounter regardless of patient prioritization. This methodological difference limits the direct comparability of the two datasets.

A closer point of comparison is provided by a retrospective analysis by Narayanan et al., also conducted at MD Anderson Cancer Center (USA), which examined 473 patients. This analysis is considerably more comparable to our study, as it likewise relied on retrospective review of medical records and used a comparably broad, predefined set of RfE categories, including diet, exercise, psychological symptoms, nausea, fatigue, pain, sleep, and neuropathy (107). The demographic profile of this cohort was comparable to ours, with 71% women (vs. 77% in our cohort), 42% breast cancer (vs. 46%), and 31% metastatic disease (vs. 41%). Given this methodological and demographic alignment, the overall symptom spectrum identified corresponds closely to the RfE recorded in our cohort, suggesting that once comparable assessment methods are applied, cancer patients’ underlying support needs across both settings are broadly similar. However, the range of recommended interventions in the Narayanan study was considerably narrower than in our study, comprising acupuncture, oncology massage, psychology referral, and mind-body therapies, whereas CCC-Integrativ consultations offered a substantially broader spectrum of NPI, NNI, and MT, 9,045 CIH Interventions in total, tailored to individual patient needs, averaging 15 specific recommendations per patient.

A broader perspective on the European IO landscape is offered by Rossi et al. (2014), who did not examine individual patient encounters but instead surveyed representatives of European centers providing integrative oncology care regarding which CIH interventions were offered (108). Across these centers, the most frequently offered modalities were acupuncture (55.3%), homeopathy (40.4%), herbal medicine (38.3%), and traditional Chinese medicine (36.2%), followed by anthroposophic medicine (21.3%) and homotoxicology (12.8%), alongside a range of other therapies (63.8%). These interventions were primarily applied to reduce adverse reactions to chemo-/radiotherapy (23.9%), particularly nausea and vomiting (13.4%) and leukopenia (5%), as well as to alleviate pain and fatigue (10.9%), side effects of iatrogenic menopause (8.8%), anxiety and depression (5.9%), gastrointestinal disorders (5%), and sleep disturbances and neuropathy (3.8% each). While this center-level perspective does not allow for direct comparison with individual patient RfE, it illustrates that the therapeutic priorities identified across European IO centers, symptom relief related to treatment side effects, broadly align with the RfE spectrum observed in our cohort, although both the frequency of RfE and the specific CIH interventions applied differ considerably between the two settings.

The most striking difference in our international comparison is the apparent absence of naturopathic nursing interventions (NNI) in the international studies reviewed (106, 107). This may point to a group of interventions that is comparatively specific to the German setting, whose potential benefit and transferability to other international contexts warrant further research. This intercultural distinction is further supported by a smaller study by Braun et al., which examined 48 patients attending an integrative oncology consultation at a German gynecological center (109). Despite being conducted exclusively by physicians, this consultation likewise reported NNI among the recommended interventions.

Taken together, these comparisons suggest that while the underlying RfE of cancer patients seeking integrative oncology support appear broadly comparable across settings once similar assessment methods are applied, the specific spectrum, depth, and interprofessional scope of recommended CIH interventions differ notably. Most notably, the prominent role of naturopathic nursing care represents a distinctive feature of our CCC-Integrativ model, one whose broader applicability and potential value for other healthcare systems merits further international investigation.

### 4.4 Strengths and limitations

Methodologically, the study falls within the scope of health services research, employing a highly practical approach at the interface between outpatient clinical care and research. To address the study aims, a qualitative content analysis with a subsequent quantitative evaluation was chosen. Deductive coding based on an established guideline enabled a structured documentation of the counseling content, while inductive expansions ensured a high degree of openness and fine granularity. Consequently, this methodology provided a realistic reflection of the actual clinical scenarios encountered during consultation hours. Analyzing the consultation letters written by the designated experts of the counseling teams enhanced content validity; however, it may also have introduced a confirmation bias.

Several limitations of this investigation must be noted, as they could constrain the interpretation of the results. Methodologically, the Delphi process used to standardize the counseling content may be susceptible to selection and consensus biases. Furthermore, multiple coders across four sites analyzed the consultation letters, rendering a systematic assessment of intercoder reliability infeasible and potentially amplifying subjective distortions (i.e., observer bias). Retrospective categorization also allowed for interpretative flexibility. Moreover, assigning data to the RfE of the ICPC-3 remains challenging, as this process can be imprecise due to the absence of standardized criteria, thereby limiting comparability with existing literature data.

The generalizability of our findings to the broader cancer population may be limited due to characteristics of the study sample. The cohort was predominantly female, relatively young, well-educated, and included a high proportion of breast cancer patients. While this reflects known utilization patterns of complementary therapies (67, 68), it inherently restricts the generalizability of the findings to broader oncology populations. Due to the recruitment methods used in the CCC-Integrativ study, a selection bias cannot be ruled out, as predominantly motivated and actively help-seeking patients were included in the intervention group (51). A more substantial limitation concerns the intercultural transferability of these findings. Certain interventions – particularly naturopathic nursing interventions (NNI), but also anthroposophic medicinal products – are predominantly available, established, and applied within German-speaking countries, reflecting local traditions of naturopathy. Consequently, the generalizability of these specific recommendations to other cultural or healthcare contexts internationally may be limited. Furthermore, the specific training and qualification of the respective professional groups involved in delivering these interventions represent an additional limitation, as such specialized education (e.g., in naturopathic nursing, acupressure, anthroposophical remedies) is not uniformly available across countries or healthcare systems, further constraining the transferability of the CCC-Integrativ counseling model. We attempted to address this challenge through a standardized training program for all participating members of the counseling teams, thereby ensuring a comparable level of competence across the four study sites.

Although this study demonstrated that patients received a wide variety of recommendations and interventions, the current analysis does not allow for conclusions regarding the actual implementation, adherence to, or effectiveness of these measures. However, the overall effectiveness of the CCC-Integrativ intervention on patient activation has already been published by Valentini et al. (51); the analysis of its effectiveness on fatigue, quality of life, and other relevant patient-reported outcomes is still forthcoming and will be reported in a separate publication. Dürsch et al. showed in their qualitative study with 38 patients that participants reported integrating a significant portion of the CIH interventions from the counseling sessions into their daily lives (63). Nonetheless, expert consensus does not represent evidence equivalent to randomized controlled trials, even though it serves as a complementary knowledge source within modern evidence-based practice. Furthermore, three consultations over a three-month period represent a relatively short timeframe for the sustainable adoption of lifestyle modifications and mind-body interventions. Finally, while some of the recommended measures were based on guidelines and clinical studies, robust evidence extending beyond expert consensus is lacking for others, particularly NNI. Their clinical effectiveness should therefore be systematically evaluated in future studies.

A major strength of this study lies in its large and robust dataset, which includes 603 patients and 1,693 consultation letters across four large study sites. The classification of RfE was based on the ICPC-3, which, compared to the International Classification of Diseases (ICD) framework, offers the advantage of a person-centered approach, thereby adequately reflecting the scenario in primary care (110–112).

Patients were counseled using a comprehensive, multimodal approach that encompassed lifestyle advice and CIH interventions – including NPI, NNI, and MT – supported by extensive informational materials and referrals to other professional providers within conventional oncological care. This demonstrates that CIH counseling not only addresses key symptoms in cancer patients but also serves as an essential guidance tool. This guiding role is of paramount importance, as it helps patients navigate the vast array of information and available options to find the most suitable and tailored interventions within their challenging clinical situation.

In contrast to the time constraints of traditional oncology consultations (70), patients in this study received a comprehensive one-hour initial consultation and two 30-minute follow-up sessions, providing sufficient time for in-depth discussions. The extracted recommendations largely aligned with international guidelines. Where interventions extended beyond these guidelines, their use was supported by preliminary evidence and systematically evaluated expert consensus. Consequently, this approach provided a broad spectrum of interventions for common burdensome symptoms in cancer care, effectively addressing unmet patient needs (71).

### 4.5 Implications for research and practice

The findings suggest that CCC-Integrativ counseling provides a feasible framework for comprehensive, patient-centered outpatient care, bridging evidence-based standards with patients’ preferences (74, 113). Key components of this approach include the broad implementation of guideline-based recommendations, the provision of evidence-informed and consensus-based strategies, and a strong emphasis on enhancing patient self-efficacy. Furthermore, it actively promotes lifestyle modifications, NPI, and NNI, all delivered through a highly collaborative, interprofessional framework.

Thus, CCC-Integrativ counseling has the potential to bridge a critical gap in outpatient oncology care. It addresses common and challenging symptoms, such as cancer-related fatigue, chemotherapy-induced peripheral neuropathy (CIPN), and sleep disturbances, which can impose substantial acute and long-term burdens on cancer patients and survivors. Furthermore, structured integrative oncology counseling may play an essential navigational role within outpatient care settings.

Overall, the present findings highlight the potential of integrative, interprofessional outpatient models for delivering holistic oncological care and provide a valuable foundation for their implementation. Future research should focus on the systematic evaluation of the clinical effectiveness of the recommended interventions – particularly those for which evidence remains limited – the implementation of existing evidence into diverse outpatient settings, and the long-term sustainability of this approach.

## Data Availability

The datasets presented in this article are not readily available because of the legal and ethical restrictions protecting patient privacy under the General Data Protection Regulation (GDPR). The raw text data (consultation letters) contain highly sensitive clinical information and cannot be shared. However, anonymized data are available from the corresponding author upon reasonable request. Requests to access the datasets should be directed to Andreas.Schmitt@med.uni-tuebingen.de.

## Supplementary material

SUPPLEMENTARY TABLE 1 Coding Guide

SUPPLEMENTARY TABLE 2 Code Sheets. **Code sheet Reasons for Encounter (RfE). Code sheet Non-Pharmacological Interventions (NPI). Code sheet Naturopathic Nursing Interventions (NNI). Code sheet Medicines and Teas (MT)**

SUPPLEMENTARY TABLE 3 Specific Reasons for Encounter by Treatment Intent

SUPPLEMENTARY TABLE 4 Recommended CIH Interventions by Treatment Intent

SUPPLEMENTARY TABLE 5 Specific Reasons for Encounter by Cancer Diagnosis

SUPPLEMENTARY TABLE 6 Recommended CIH Interventions by Cancer Diagnosis

## References

[1] Witt CM Balneaves LG Cardoso MJ Cohen L Greenlee H Johnstone P. A comprehensive definition for integrative oncology. J Natl Cancer Inst Monogr. (2017) 2017(52):lgx012. 10.1093/jncimonographs/lgx012 29140493

[2] Huebner J Prott FJ Micke O Muecke R Senf B Dennert G. Online survey of cancer patients on complementary and alternative medicine. Oncol Res Treat. (2014) 37(6):304–8. 10.1159/000362616 24903760

[3] Grimm D Mathes S Woelber L Van Aken C Schmalfeldt B Mueller V. Demand for integrative medicine among women with breast and gynecological cancer: a multicenter cross-sectional study in southern and northern Germany. Arch Gynecol Obstet. (2021) 303(5):1315–30. 10.1007/s00404-020-05880-0 33263787 PMC8053147

[4] Horneber M Bueschel G Dennert G Less D Ritter E Zwahlen M. How many cancer patients use complementary and alternative medicine: a systematic review and metaanalysis. Integr Cancer Ther. (2012) 11(3):187–203. 10.1177/1534735411423920 22019489

[5] Rombouts MD Karg RMY Raddjoe SSP Crul M. Over 40% of cancer patients use complementary and alternative medications while receiving anticancer treatment. Eur J Hosp Pharm. (2025) 32(4):348–53. 10.1136/ejhpharm-2024-004264 39710493 PMC12322446

[6] Jermini M Dubois J Rodondi P-Y Zaman K Buclin T Csajka C. Complementary medicine use during cancer treatment and potential herb-drug interactions from a cross-sectional study in an academic centre. Sci Rep. (2019) 9(1):5078. 10.1038/s41598-019-41532-3 30911084 PMC6434040

[7] Arthur KN Belliard JC Hardin SB Knecht K Chen C-S Montgomery S. Reasons to use and disclose use of complementary medicine use - an insight from cancer patients. Cancer Clin Oncol. (2013) 2(2):81–92. 10.5539/cco.v2n2p81 26640610 PMC4667734

[8] Onkologie L. S3-Leitlinie Komplementärmedizin in der Behandlung von onkologischen PatientInnen, Langversion 2.0, 2024, AWMF-Registernummer: 032-055OL. 2024 (2024).

[9] Cardoso F Paluch-Shimon S Senkus E Curigliano G Aapro MS André F. 5th ESO-ESMO international consensus guidelines for advanced breast cancer (ABC 5). Ann Oncol. (2020) 31(12):1623–49. 10.1016/j.annonc.2020.09.010 32979513 PMC7510449

[10] Lyman GH Greenlee H Bohlke K Bao T DeMichele AM Deng GE. Integrative therapies during and after breast cancer treatment: aSCO endorsement of the SIO clinical practice guideline. J Clin Oncol. (2018) 36(25):2647–55. 10.1200/JCO.2018.79.2721 29889605 PMC13123314

[11] Carlson LE Ismaila N Addington EL Asher GN Bradt J Mehta A. Integrative oncology care of symptoms of anxiety and depression in adults with cancer: sIO-ASCO guideline summary and Q&A. JCO Oncol Pract. (2023) 19(10):847–51. 10.1200/OP.23.00358 37582242

[12] Schmidt ME Goldschmidt S Kreutz C Müller J Schneeweiss A May AM. Effects of aerobic or resistance exercise during neoadjuvant chemotherapy on tumor response and therapy completion in women with breast cancer: the randomized controlled BENEFIT trial. J Sport Health Sci. (2025) 14:101064. 10.1016/j.jshs.2025.101064 40447136 PMC12395146

[13] Oberoi S Robinson PD Cataudella D Culos-Reed SN Davis H Duong N. Physical activity reduces fatigue in patients with cancer and hematopoietic stem cell transplant recipients: a systematic review and meta-analysis of randomized trials. Crit Rev Oncol Hematol. (2018) 122:52–9. 10.1016/j.critrevonc.2017.12.011 29458789

[14] Campbell K Winters-Stone K Wiskemann J May A Schwartz A Courneya K. Exercise guidelines for cancer survivors: consensus statement from international multidisciplinary roundtable. Med Sci Sports Exercise. (2019) 51(11):2375–90. 10.1249/MSS.0000000000002116 PMC8576825 31626055

[15] Nuñez de Arenas-Arroyo S Cavero-Redondo I Torres-Costoso A Reina-Gutiérrez S Lorenzo-García P Martínez-Vizcaíno V. Effects of exercise interventions to reduce chemotherapy-induced peripheral neuropathy severity: a meta-analysis. Scand J Med Sci Sports. (2023) 33(7):1040–53. 10.1111/sms.14360 36972017

[16] Garcia L Pearce M Abbas A Mok A Strain T Ali S. Non-occupational physical activity and risk of cardiovascular disease, cancer and mortality outcomes: a dose-response meta-analysis of large prospective studies. Br J Sports Med. (2023) 57(15):979–89. 10.1136/bjsports-2022-105669 36854652 PMC10423495

[17] Friedenreich CM Stone CR Cheung WY Hayes SC. Physical activity and mortality in cancer survivors: a systematic review and meta-analysis. JNCI Cancer Spectr. (2020) 4(1):pkz080. 10.1093/jncics/pkz080 32337494 PMC7050161

[18] Courneya KS Vardy JL O’Callaghan CJ Gill S Friedenreich CM Wong RKS. Structured exercise after adjuvant chemotherapy for colon cancer. N Engl J Med. (2025) 393(1):13–25. 10.1056/NEJMoa2502760 40450658

[19] Ungvari Z Fekete M Varga P Lehoczki A Munkácsy G Fekete JT. Association between red and processed meat consumption and colorectal cancer risk: a comprehensive meta-analysis of prospective studies. Geroscience. (2025) 47(3):5123–40. 10.1007/s11357-025-01646-1 40210826 PMC12181564

[20] Huang T Yang B Zheng J Li G Wahlqvist ML Li D. Cardiovascular disease mortality and cancer incidence in vegetarians: a meta-analysis and systematic review. Ann Nutr Metab. (2012) 60(4):233–40. 10.1159/000337301 22677895

[21] Chen S Chen Y Ma S Zheng R Zhao P Zhang L. Dietary fibre intake and risk of breast cancer: a systematic review and meta-analysis of epidemiological studies. Oncotarget. (2016) 7(49):80980–9. 10.18632/oncotarget.13140 27829237 PMC5348370

[22] Zademohammadi F Sasanfar B Toorang F Mozafarinia M Salehi-Abargouei A Zendehdel K. Dietary soluble, insoluble, and total fiber intake and their dietary sources in association with breast cancer. BMC Public Health. (2024) 24(1):2560. 10.1186/s12889-024-19861-4 39300417 PMC11414166

[23] Arends J Bertz H Bischoff SC Fietkau R Herrmann HJ Holm E. S3-Leitlinie Klinische Ernährung in der Onkologie. (2015). Available online at: http://www.thieme-connect.de/products/ejournals/pdf/10.1055/s-0035-1552741.pdf (Accessed December 02, 2026).

[24] Haller H Winkler MM Klose P Dobos G Kümmel S Cramer H. Mindfulness-based interventions for women with breast cancer: an updated systematic review and meta-analysis. Acta Oncol. (2017) 56(12):1665–76. 10.1080/0284186X.2017.1342862 28686520

[25] Agarwal RP Maroko-Afek A. Yoga into cancer care: a review of the evidence-based research. Int J Yoga. (2018) 11(1):3–29. 10.4103/ijoy.IJOY_42_17 29343927 PMC5769195

[26] Cramer H Lauche R Klose P Lange S Langhorst J Dobos GJ. Yoga for improving health-related quality of life, mental health and cancer-related symptoms in women diagnosed with breast cancer. Cochrane Database Syst Rev. (2017) 2017(1):Cd010802. 10.1002/14651858.CD010802.pub2 PMC6465041 28045199

[27] Wayne PM Lee MS Novakowski J Osypiuk K Ligibel J Carlson LE. Tai chi and qigong for cancer-related symptoms and quality of life: a systematic review and meta-analysis. J Cancer Surviv. (2018) 12(2):256–67. 10.1007/s11764-017-0665-5 29222705 PMC5958892

[28] Liu P Li L Xu D Xin S Hu N Li C. Acupuncture for cancer-related insomnia: systematic review and meta-analysis of randomised controlled trials. BMJ Support Palliat Care. (2024) 14(4):378–91. 10.1136/spcare-2024-005051 39384192

[29] He Y Guo X May BH Zhang AL Liu Y Lu C. Clinical evidence for association of acupuncture and acupressure with improved cancer pain: a systematic review and meta-analysis. JAMA Oncol. (2020) 6(2):271–8. 10.1001/jamaoncol.2019.5233 31855257 PMC6990758

[30] Tian H Chen Y Sun M Huang L Xu G Yang C. Acupuncture therapies for cancer-related fatigue: a Bayesian network meta-analysis and systematic review. Front Oncol. (2023) 13:1071326. 10.3389/fonc.2023.1071326 37051548 PMC10083363

[31] Hershman DL Unger JM Greenlee H Capodice JL Lew DL Darke AK. Effect of acupuncture vs sham acupuncture or waitlist control on joint pain related to aromatase inhibitors among women with early-stage breast cancer: a randomized clinical trial. JAMA. (2018) 320(2):167–76. 10.1001/jama.2018.8907 29998338 PMC6583520

[32] Zhang X Hou W Pu F Wang X Wang Y Yang M. Acupuncture for cancer-related conditions: an overview of systematic reviews. Phytomedicine. (2022) 106:154430. 10.1016/j.phymed.2022.154430 36099656

[33] Valentini J. Can traditional Chinese medicine help cancer patients? Rev Med Suisse. (2016) 12(503):213–5. 10.53738/REVMED.2016.12.503.0213 26939187

[34] Stolz R Klafke N Kröger B Boltenhagen U Kaltenbach A Heine R. Generierung von evidenz zu naturheilkundlichen pflegeinterventionen in der onkologie–ein systematisches verfahren. Zeitschrift für Evidenz, Fortbildung und Qualität im Gesundheitswesen. (2021) 166:1–7. 10.1016/j.zefq.2021.08.005 34563466

[35] Raiber L Raff C Thiele J Kramer K. Integrative nursing interventions for cancer-related symptoms in oncology inpatients: results of a descriptive pilot study. Integr Cancer Ther. (2024) 23:15347354241239930. 10.1177/15347354241239930 38680047 PMC11057344

[36] Stolz R Kaschdailewitsch E Kröger B Noack B Mahler C. Evidenzbasierte naturheilkundliche pflegeinterventionen in der schmerztherapie. Der Schmerz. (2023) 37(5):336–43. 10.1007/s00482-023-00705-w 37039897 PMC10511583

[37] Winkler M Breitkreuz T Brust J Frenzel S Gottfried J Heyl W. Expert-guided approaches to complementary interventions for common side effects of cancer therapies: a practice-based perspective from integrative oncology centers in Baden-Württemberg, Germany. Front Oncol. (2025) 15:1667298. 10.3389/fonc.2025.1667298 41278285 PMC12631479

[38] Trümper L Mößner U Schäfer N Schönfeld S. Personalsituation Pflege Auswertung der Umfrage zur Pflegepersonalausstattung in hämatologischen und onkologischen Krankenhausbereichen, D.D.G.f. Hämatologie and u.M.O.e. V., editors (2021).

[39] Kaiser U Kaiser F Damnali G Vehling-Kaiser U. Die nicht-ärztliche onkologische und hämatologische pflegesprechstunde. Die Onkologie. (2024) 30(8):729–34. 10.1007/s00761-024-01545-8

[40] Kohler S Alig B. Komplementäre pflege in der onkologie. Die Urologie. (2023) 62(11):1217–20. 10.1007/s00120-023-02217-y 37878042 PMC10630179

[41] Hesmert D Klocke C Stolz R Huber R Samstag Y Hübner K. Exploring the gap: attitudes, knowledge, and training needs in complementary and integrative medicine among healthcare professionals at German university hospitals. Front Med (Lausanne). (2024) 11:1408653. 10.3389/fmed.2024.1408653 38784234 PMC11111851

[42] Trimborn A Senf B Muenstedt K Buentzel J Micke O Muecke R. Attitude of employees of a university clinic to complementary and alternative medicine in oncology. Ann Oncol. (2013) 24(10):2641–5. 10.1093/annonc/mdt299 23942776

[43] Ben-Arye E Shulman B Eilon Y Woitiz R Cherniak V Shalom Sharabi I. Attitudes among nurses toward the integration of complementary medicine into supportive cancer care. Oncol Nurs Forum. (2017) 44(4):428–34. 10.1188/17.ONF.428-434 28632238

[44] Klafke N Mahler C Von Hagens C Uhlmann L Bentner M Schneeweiss A. The effects of an integrated supportive care intervention on quality of life outcomes in outpatients with breast and gynecologic cancer undergoing chemotherapy: results from a randomized controlled trial. Cancer Med. (2019) 8(8):3666–76. 10.1002/cam4.2196 31115192 PMC6639168

[45] Balouchi A Mahmoudirad G Hastings-Tolsma M Shorofi SA Shahdadi H Abdollahimohammad A. Knowledge, attitude and use of complementary and alternative medicine among nurses: a systematic review. Complement Ther Clin Pract. (2018) 31:146–57. 10.1016/j.ctcp.2018.02.008 29705447

[46] Kaiser F Vehling-Kaiser U Hermes-Moll K Walawgo T Baumann W. Feasibility of nurse consultation in oral tumor therapy: a web-based survey among physicians and nonmedical specialists. Oncol Res Treat. (2019) 42(9):448–57. 10.1159/000501725 31336372

[47] Song HJ Seo H-J Choi EJ Lee JS Choi Y. Nursing care services to address unmet supportive care needs among cancer survivors: a systematic review. J Cancer Surviv. (2024) 20:427–52. 10.1007/s11764-024-01661-9 39240428

[48] Song Y Li J Feng Y Li S Hong J. Actual needs and perceived supply of nursing supportive care among patients with cancer in mainland China: a cross-sectional study. Jpn J Nurs Sci. (2022) 19(1):e12449. 10.1111/jjns.12449 34498402

[49] Lukewich J Martin-Misener R Norful AA Poitras M-E Bryant-Lukosius D Asghari S. Effectiveness of registered nurses on patient outcomes in primary care: a systematic review. BMC Health Serv Res. (2022) 22(1):740. 10.1186/s12913-022-07866-x 35659215 PMC9166606

[50] Homberg A Stock-Schröer B. Interprofessional education on complementary and integrative medicine. Clin Teach. (2021) 18(2):152–7. 10.1111/tct.13280 33029879

[51] Valentini J Froehlich D Roesel I Stolz R Mahler C Martus P. Enhancing patient activation: a controlled implementation study of an interprofessional evidence-based counseling program for complementary and integrative healthcare in cancer patients (‘CCC-integrativ’). Front Med. (2024) 18(6):1013–25. 10.1007/s11684-024-1097-z 39542987

[52] Cramer H Schleinzer A Bilc MI. Interprofessional collaboration in integrative and complementary medicine. J Integr Complement Med. (2025) 31(7):589–90. 10.1089/jicm.2025.0417 40522741

[53] Ben-Arye E Samuels N Goldstein LH Mutafoglu K Omran S Schiff E. Potential risks associated with traditional herbal medicine use in cancer care: a study of middle eastern oncology health care professionals. Cancer. (2016) 122(4):598–610. 10.1002/cncr.29796 26599199

[54] Posadzki P Watson LK Ernst E. Adverse effects of herbal medicines: an overview of systematic reviews. Clin Med (Lond). (2013) 13(1):7–12. 10.7861/clinmedicine.13-1-7 23472485 PMC5873713

[55] Wolf CPJG Rachow T Ernst T Hochhaus A Zomorodbakhsch B Foller S. Complementary and alternative medicine (CAM) supplements in cancer outpatients: analyses of usage and of interaction risks with cancer treatment. J Cancer Res Clin Oncol. (2022) 148(5):1123–35. 10.1007/s00432-021-03675-7 34228225 PMC9016053

[56] Johnson SB Park HS Gross CP Yu JB. Complementary medicine, refusal of conventional cancer therapy, and survival among patients with curable cancers. JAMA Oncol. (2018) 4(10):1375–81. 10.1001/jamaoncol.2018.2487 30027204 PMC6233773

[57] King N Balneaves LG Levin GT Nguyen T Nation JG Card C. Surveys of cancer patients and cancer health care providers regarding complementary therapy use, communication, and information needs. Integr Cancer Ther. (2015) 14(6):515–24. 10.1177/1534735415589984 26069277

[58] Chang KH Brodie R Choong MA Sweeney KJ Kerin MJ. Complementary and alternative medicine use in oncology: a questionnaire survey of patients and health care professionals. BMC Cancer. (2011) 11:196. 10.1186/1471-2407-11-196 21609461 PMC3123324

[59] Chang HY Chang HL. A review of nurses’ knowledge, attitudes, and ability to communicate the risks and benefits of complementary and alternative medicine. J Clin Nurs. (2015) 24(11-12):1466–78. 10.1111/jocn.12790 25727902

[60] Ng JY Shah AQ Narr LA Qian A Cramer H. Physicians' knowledge and experiences of counseling on complementary, alternative, and integrative medicine in cancer care: a qualitative systematic review. Curr Oncol Rep. (2026) 28(1):49. 10.1007/s11912-026-01772-9 42105165 PMC13157459

[61] Valentini J Fröhlich D Stolz R Mahler C Martus P Klafke N. Interprofessional evidence-based counselling programme for complementary and integrative healthcare in patients with cancer: study protocol for the controlled implementation study CCC-integrativ. BMJ Open. (2022) 12(2):e055076. 10.1136/bmjopen-2021-055076 35149568 PMC8845169

[62] Mahler C Joos S. Integrative Interventionen in der Onkologie: Ein interprofessionelles Praxis-und Beratungshandbuch. Bern: Hogrefe AG (2025).

[63] Dürsch H Boltenhagen U Mahler C Joos S Joachim S Klafke N. A qualitative investigation of factors influencing the integration of complementary and integrative healthcare recommendations in the daily lives of patients with cancer. Integr Cancer Ther. (2024) 23:15347354241252195. 10.1177/15347354241252195 PMC11143848 38812440

[64] Klafke N Bossert J Boltenhagen U Froehlich D Mahler C Joos S. Counseling lifestyle medicine in oncology: a qualitative analysis of interprofessional patient-nurse-physician interactions. Patient Educ Couns. (2024) 127:108352. 10.1016/j.pec.2024.108352 38905751

[65] Mayring P GmbH JB KG C. Qualitative Inhaltsanalyse: Grundlagen und Techniken. Weinheim: Beltz (2022).

[66] Napel HT Kees VB. ICPC-3 User Manual, Guidance on how to code health data in Primary Health Care 2022 (2022).

[67] Wortmann JK Bremer A Eich HT Wortmann HPK Schuster A Fühner J. Use of complementary and alternative medicine by patients with cancer: a cross-sectional study at different points of cancer care. Med Oncol. (2016) 33(7):78. 10.1007/s12032-016-0790-4 27300549

[68] Witt C. Komplementärmedizin: worüber patientinnen informiert werden sollten. Kompass Onkologie. (2022) 9(2):74–5. 10.1159/000524882

[69] Pierce JP Stefanick ML Flatt SW Natarajan L Sternfeld B Madlensky L. Greater survival after breast cancer in physically active women with high vegetable-fruit intake regardless of obesity. J Clin Oncol. (2007) 25(17):2345–51. 10.1200/JCO.2006.08.6819 17557947 PMC2274898

[70] Guy GP Richardson LC. Visit duration for outpatient physician office visits among patients with cancer. J Oncol Pract. (2012) 8(3 Suppl):2s–8. 10.1200/JOP.2011.000493 22942822 PMC3348598

[71] Fridman I Johnson SB Derry-Vick HM. When limited clinical time with patients meets unlimited online information. JMIR Cancer. (2025) 11:e79031. 10.2196/79031 41004797 PMC12514413

[72] Fabi A Bhargava R Fatigoni S Guglielmo M Horneber M Roila F. Cancer-related fatigue: eSMO clinical practice guidelines for diagnosis and treatment. Ann Oncol. (2020) 31(6):713–23. 10.1016/j.annonc.2020.02.016 32173483

[73] Mustian K Lacchetti C Zick S Bower JE. Management of fatigue in adult survivors of cancer: american society of clinical oncology - society for integrative oncology (ASCO-SIO) guideline update clinical insights. JCO Oncol Pract. (2024) 20(12):1575–9. 10.1200/OP.24.00372 38954779

[74] Sackett DL Rosenberg WMC Gray JAM Haynes RB Richardson WS. Evidence based medicine: what it is and what it isn't. Br Med J. (1996) 312(7023):71–2. 10.1136/bmj.312.7023.71 PMC2349778 8555924

[75] Dürsch H Boltenhagen U Mahler C Joos S Szecsenyi J Klafke N. A qualitative analysis of cancer Patients’ perceptions of an interprofessional counseling service on complementary and integrative healthcare. Qual Health Res. (2024) 34(12):1132–46. 10.1177/10497323241231530 PMC11528944 38441438

[76] Committee on Herbal Medicinal Products. H., COMMUNITY HERBAL MONOGRAPH ON PSYLLIUM SEED (PLANTAGO AFRA ET PLANTAGO INDICA, SEMEN) (2005).

[77] (HMPC), C.o.H.M.P. Community herbal monograph on Lavandula angustifolia Miller, aetheroleum (2012).

[78] (HMPC), C.o.H.M.P. European Union herbal monograph on Valeriana officinalis L., radix (2016).

[79] Artan Y Sezgin G Bulut İ Yildirim Y. The role of disease duration in the use of complementary and integrative medicine for cancer-related fatigue: a cross-sectional study. Support Care Cancer. (2025) 33(4):303. 10.1007/s00520-025-09367-z 40111525 PMC11926054

[80] Han C Li N Wang X Zhuang Z Cao Q Wang S. Sleep disorders in cancer: interactions and intrinsic links. Front Oncol. (2025) 15:1535442. 10.3389/fonc.2025.1535442 40740866 PMC12307220

[81] Strollo SE Fallon EA Gapstur SM Smith TG. Cancer-related problems, sleep quality, and sleep disturbance among long-term cancer survivors at 9-years post diagnosis. Sleep Med. (2020) 65:177–85. 10.1016/j.sleep.2019.10.008 32029206

[82] Truant T Porcino A Ross B Wong M Hilario C. Complementary and alternative medicine (CAM) use in advanced cancer: a systematic review. J Support Oncol. (2013) 11(3):105–13. 10.12788/j.suponc.0011 24400390

[83] Ma Y He B Jiang M Yang Y Wang C Huang C. Prevalence and risk factors of cancer-related fatigue: a systematic review and meta-analysis. Int J Nurs Stud. (2020) 111:103707. 10.1016/j.ijnurstu.2020.103707 32920423

[84] Al Maqbali M Al Sinani M Al Naamani Z Al Badi K Tanash M'I. Prevalence of fatigue in patients with cancer: a systematic review and meta-analysis. J Pain Symptom Manage. (2021) 61(1):167–189.e14. 10.1016/j.jpainsymman.2020.07.037 32768552

[85] Henry DH Viswanathan HN Elkin EP Traina S Wade S Cella D. Symptoms and treatment burden associated with cancer treatment: results from a cross-sectional national survey in the U.S. Support Care Cancer. (2008) 16(7):791–801. 10.1007/s00520-007-0380-2 18204940

[86] Mattar M Umutoni F Hassan MA Wamburu MW Turner R Patton JS. Chemotherapy-induced peripheral neuropathy: a recent update on pathophysiology and treatment. Life (Basel). (2024) 14(8):991. 10.3390/life14080991 39202733 PMC11355765

[87] D’Souza RS Saini C Hussain N Javed S Prokop L Her YF. Global estimates of prevalence of chronic painful neuropathy and moderate-to-severe neuropathy among patients with chemotherapy-induced peripheral neuropathy: a systematic review and meta-analysis of data from 29 countries between 2000 and 2024. Regional Anesthesia & Pain Medicine. (2025) 51:rapm-2024-106229. 10.1136/rapm-2024-106229 39880412

[88] Jordan K. German Cancer Society, German Cancer Aid, and AWMF. S3-Guideline Supportive Therapy in Oncological Patients - Long Version 2.0. (2025). Available online at: https://www.leitlinienprogramm-onkologie.de (Accessed May 28, 2026).

[89] Chen M-Y Zheng W-Y Liu Y-F Li X-H Lam MI Su Z. Global prevalence of poor sleep quality in cancer patients: a systematic review and meta-analysis. Gen Hosp Psychiatry. (2024) 87:92–102. 10.1016/j.genhosppsych.2023.12.004 38382421

[90] Al Maqbali M Al Sinani M Alsayed A Gleason AM. Prevalence of sleep disturbance in patients with cancer: a systematic review and meta-analysis. Clin Nurs Res. (2022) 31(6):1107–23. 10.1177/10547738221092146 35484919 PMC9266067

[91] Snijders R Brom L Theunissen M Van Den Beuken-Van Everdingen M. Update on prevalence of pain in patients with cancer 2022: a systematic literature review and meta-analysis. Cancers (Basel). (2023) 15(3):591. 10.3390/cancers15030591 36765547 PMC9913127

[92] Bower JE. Cancer-related fatigue–mechanisms, risk factors, and treatments. Nat Rev Clin Oncol. (2014) 11(10):597–609. 10.1038/nrclinonc.2014.127 25113839 PMC4664449

[93] Zick SM Chen D Harris RE Kruger G Runyon A Sen A. Self-acupressure for fatigue in patients surviving ovarian cancer: a randomized clinical trial. JAMA Netw Open. (2026) 9(2):e2556357. 10.1001/jamanetworkopen.2025.56357 41642626 PMC12878437

[94] Chou H-C Konara Mudiyanselage SP Tang C-C Chen S-C Fang S-Y Lin M-F. Comparative effectiveness of acupoint stimulation therapies for cancer-related fatigue: a network meta-analysis. Worldviews Evid Based Nurs. (2026) 23(1):e70104. 10.1111/wvn.70104 41618084

[95] Pelzer F Loef M Martin DD Baumgartner S. Cancer-related fatigue in patients treated with mistletoe extracts: a systematic review and meta-analysis. Support Care Cancer. (2022) 30(8):6405–18. 10.1007/s00520-022-06921-x 35239008 PMC9213316

[96] Mahler C Stolz R. Pflege in der integrativen medizin. In: eds. B. Brinkhaus, T. Esch. Integrative Medizin und Gesundheit. Berlin: MWV Medizinisch Wissenschaftliche Verlagsgesellschaft (2021). p. 265–78.

[97] Mahler C. Naturheilkundliche pflegemaßnahmen in der onkologie. Pflegezeitschrift. (2019) 72(3):56–9. 10.1007/s41906-019-0013-7

[98] Cheung DST Yeung WF Chau PH Chan SY Chan WL Yeh CH. Improving chemotherapy-induced peripheral neuropathy in cancer patients using a combined qigong and self-administered acupressure intervention: a randomized controlled trial. ESMO Open. (2025) 10(9):105565. 10.1016/j.esmoop.2025.105565 40840230 PMC12529305

[99] Jafari M Shabani F Fathollahi MS Barghi TS Peighambari SB Zargaz SEH. Effect of acupressure on pain and sleep quality of patients with cancer after undergoing surgery admitted to the intensive care unit: a single-blind randomized clinical trial. Iran J Nurs Midwifery Res. (2025) 30(4):531–7. 10.4103/ijnmr.ijnmr_368_23 40832492 PMC12360763

[100] Yang L-H Li S-Y Yang Y Yang S-N Guo H-L Hu M-M. Effectiveness of acupressure in managing the pain-fatigue-sleep disturbance-depression symptom cluster in patients with cancer: a systematic review and meta-analysis of randomized controlled trials. J Integr Complement Med. (2025) 31(10):860–75. 10.1089/jicm.2024.0914 40569723

[101] Khosravaninezhad Y Moshfeghinia R Liaghat L Ghazipoor H Kaheni Y Javidan A. Effect of acupressure and acupuncture on sleep quality in cancer patients with insomnia: a systematic review and meta-analysis. Explore (NY). (2025) 21(4):103192. 10.1016/j.explore.2025.103192 40449465

[102] Barton DL Atherton PJ Bauer BA Moore DF Mattar BI LaVasseur BI. The use of Valeriana officinalis (valerian) in improving sleep in patients who are undergoing treatment for cancer: a phase III randomized, placebo-controlled, double-blind study (NCCTG trial, N01C5). J Support Oncol. (2011) 9(1):24–31. 10.1016/j.suponc.2010.12.008 21399726 PMC3052692

[103] Mardani A Maleki M Hanifi N Borghei Y Vaismoradi M. A systematic review of the effect of lavender on cancer complications. Complement Ther Med. (2022) 67:102836. 10.1016/j.ctim.2022.102836 35462027

[104] Donelli D Antonelli M Bellinazzi C Gensini GF Firenzuoli F. Effects of lavender on anxiety: a systematic review and meta-analysis. Phytomedicine. (2019) 65:153099. 10.1016/j.phymed.2019.153099 31655395

[105] Rother C Schnelle M. Cardiodoron® bei patienten mit schlafstörungen - ergebnisse einer prospektiven beobachtungsstudie. Complement Med Res. (2017) 24(1):15–25. 10.1159/000452236 27924793

[106] Lopez G McQuade J Cohen L Williams JT Spelman AR Fellman B. Integrative oncology physician consultations at a comprehensive cancer center: analysis of demographic, clinical and patient reported outcomes. J Cancer. (2017) 8(3):395–402. 10.7150/jca.17506 28261340 PMC5332890

[107] Narayanan S Liu W Lopez G Fellman B Reddy A Subbiah IM. Practice patterns on the incorporation of integrative medicine into the oncologic care of patients with cancer. Integr Cancer Ther. (2023) 22:15347354231213045. 10.1177/15347354231213045 37978821 PMC10657516

[108] Rossi E Vita A Baccetti S Di Stefano M Voller F Zanobini A. Complementary and alternative medicine for cancer patients: results of the EPAAC survey on integrative oncology centres in Europe. Support Care Cancer. (2015) 23(6):1795–806. 10.1007/s00520-014-2517-4 25471177

[109] Braun E-M Sklavounos P Braun BJ Wagner S Juhasz-Böss I Solomayer E-F. Integrative onkologisch-gynäkologische sprechstunde – erfahrungen des ersten jahres. Der Gynäkologe. (2020) 53(6):388–96. 10.1007/s00129-020-04596-1

[110] Hartman TC Van Ravesteijn H Lucassen P Van Boven K Van Weel-Baumgarten E Van Weel C. Why the ‘reason for encounter’ should be incorporated in the analysis of outcome of care. Br J Gen Pract. (2011) 61(593):e839–41. 10.3399/bjgp11X613269 22242223 PMC3223782

[111] Soler JK Okkes I. Reasons for encounter and symptom diagnoses: a superior description of patients’ problems in contrast to medically unexplained symptoms (MUS). Fam Pract. (2012) 29(3):272–82. 10.1093/fampra/cmr101 22308181

[112] Napel HT Van Boven K Olagundoye OA Van Der Haring E Verbeke M Härkönen M. Improving primary health care data with ICPC-3: from a medical to a person-centered perspective. Ann Fam Med. (2022) 20(4):358–61. 10.1370/afm.2830 35879074 PMC9328701

[113] Shapiro SD Logvinov I Thomas RM. Redefining evidence-based practice through patient values: a theoretical innovation for person-centered, value-based care. J Eval Clin Pract. (2026) 32(1):e70344. 10.1111/jep.70344s 41569875

