# Supplementary material for: Reasons for encounter and recommended interventions in an interprofessional integrative oncology counseling program in outpatient care: a mixed-methods analysis of 1,693 consultation letters
Source: Front Med (Lausanne). 2026 Oct 7;13:1907654. doi: 10.3389/fmed.2026.1907654 (PMC13683791; doi:10.3389/fmed.2026.1907654)
Supplement: Supplementary file 1 [file Table1.docx]

### Supplement 1: Coding Guide

| Keyword | Rule |
| --- | --- |
| Activation | Sometimes a measure was prescribed to promote ‘activation’. However, there are also very unusual symptoms such as ‘low motivation’. These can then be subsumed under categories such as activation or relaxation, depending on the context. |
| Anxiety | Anxiety is often mentioned alongside tension and inner restlessness and can, where appropriate, be grouped together so that the main symptom is used. (Inner) restlessness, however, also has a separate code. |
| General counseling | Our general lifestyle topics are exercise, nutrition and relaxation. These are recorded separately. However, they can also be listed again separately as a “symptom”, i.e. the reason for encounter, if a specific measure was recommended in relation to them. |
| Psychosocial counseling | All psycho-oncological/psychosocial support services, including the provision of leaflets or booking appointments. |
| Relaxation | This also includes stress management, |
| Fatigue | Sometimes fatigue is described using several terms, e.g. exhaustion, tiredness, weariness, etc. In such cases, the symptoms can be subsumed under fatigue. However, tiredness also exists as a separate symptom |
| Leaflets | These are likely to be very location-specific. Please consult with us. |
| Foot bath – alkaline salt | This also includes sea salt or salt |
| Skin problems | This is a very general term and is often used in this way. Specific symptoms, such as hand-foot syndrome, are listed separately. |
| Cachexia | This also includes, for example, muscle wasting, if it is serious. |
| Mucosal problems | While mucositis is a type of mucosal issue, it is a very distinct and specific condition in its own right coded separately. |
| Others | For symptoms that are not part of the oncological treatment, especially symptoms that occur only sporadically, please list them under “Others” and add a note. |
| Specific nutritional advice | If advice was given not just generally on nutrition (whole foods, vegetables, etc.), but specifically, e.g. on dietary supplements/vitamin supplementation/level testing/micronutrients, or if a leaflet on nutrition was provided or a special consultation was organized. |
| Umlauts | Do not use Ä, Ö, Ü… :-) |
| Support | If something is provided merely for “support”, the symptom can be interpreted from the context: e.g. rosemary for stimulation, lavender for relaxation or sleep disorders |
| Digestive complains | This category includes, for example: stomach ache, flatulence, non-specific abdominal complaints, abdominal cramps, stimulation of digestion |
| Additional Codes | It is very likely that additional coding requirements will arise over time. Please use provisory codes and consult with us. |
