# Supplementary material for: Reasons for encounter and recommended interventions in an interprofessional integrative oncology counseling program in outpatient care: a mixed-methods analysis of 1,693 consultation letters
Source: Front Med (Lausanne). 2026 Oct 7;13:1907654. doi: 10.3389/fmed.2026.1907654 (PMC13683791; doi:10.3389/fmed.2026.1907654)
Supplement: Supplementary file 2 [file Table2.docx]

### Supplement 2.1: Code sheet Reasons for Encounter (RfE)

| **RfE** | **Code** | **RfE** | **Code** |
| --- | --- | --- | --- |
| Activation | Akt | Elevated liver enzymes | Lbr |
| Anxiety | Ang | Lymphoedema | Lym |
| Loss of appetite | App | Mistletoe | Mis |
| Eye problems | Aug | Mucositis | Muc |
| Abdominal cramps | Bau | Tiredness | Mue |
| Stress of the children | Kin | Nail problems | Nag |
| Exercise | Bew | Dietary supplements | Nem |
| Flatulence | Bla | Scars | Nar |
| Cheomtherapy induced peripheral neuropathy | Cip | Constipation | Obs |
| Depressive mood | Dep | Oedema | Oed |
| Diarrhoea | Dur | Osteoporosis | Ost |
| Dysgeusia | Ges | Pruritus | Juc |
| Dyspnoea | Dys | Psychological stress | Psy |
| Relaxation | Ent | Dry cough | Rei |
| Vomiting | Erb | Restless legs | Res |
| Nutrition | Ern | Sleep disturbance | Scf |
| Exhaustion | Ers | Mucosal problems | Slh |
| Fatigue | Fat | Pain | Scz |
| Financial stress | Fin | Seroma | Ser |
| Questions about specific treatment methods | Fr_Sp | Heartburn | Sod |
| Hair loss | Haa | Thermoregulation disorders | Wae |
| Hand-foot syndrome | Han | Stress management | Str |
| Skin reactions | Hau | Death | Tod |
| Cerebral oedema | Hir | Sadness | Tra |
| Hot flushes | Hit | Cachexia | Kac |
| Homeopathy | Hom | Nausea | Ueb |
| Cough | Hus | Restlessness | Unr |
| Immune system | Imm | Malaise | Unw |
| Incontinence | Ink | Digestive complaints | Ver |
| Cold feet | Kal | Calf cramps | Wad |
| Concentration problems | Kon | Xerostomia | Mun |
| Headaches | Ksz | Tongue coating | Zun |
| Circulatory problems | Kre | Cystitis | Zys |
| Quality of life | Leb |  |  |

### Supplement 2.2: Code sheet Non-Pharmacological Interventions (NPI)

| **NPI** | **Code** | **NPI** | **Code** |
| --- | --- | --- | --- |
| Mindfulness-based movement therapy (general) (>=2) | Ac_Al | Balance exercises | Gl_Ue |
| Therapeutic Eurythmy | Ac_He | Massage with a spiky ball | Ma_Ig |
| Qi Gong | Ac_Qi | Seed bath | Sa_Ba |
| Tai Chi | Ac_Ta | Sensory exercises | Se_Ue |
| Yoga | Ac_Yo |  |  |
| Acupressure for anxiety | Ak_An | Link to GAED | Li_Ga |
| Acupressure for CIPN | Ak_Ci | One-third solution | Dr_Lo |
| Acupressure for diarrhoea | Ak_Di | Healing clay | Hei |
| Acupressure for fatigue | Ak_Fa | Millet | Hir |
| Acupressure for dry skin | Ak_Ha | High-calorie diet | Ho_Er |
| Acupressure for hot flushes | Ak_Hi | Moro soup | Mo_Su |
| Acupressure for itching | Ak_Ju | Specific dietary advice, nutrition leaflet, micronutrients, referral, | Sp_Er |
| Acupressure for lymphatic congestion | Ak_Ly | Exercise, sports medicine leaflet | Spo |
| Acupressure for dry mouth (DH) | Ak_Mt | Cancer Information Service | Li_Kid |
| Acupressure for mucositis | Ak_Mu | Link to Nursing Vademecum | Li_Pf |
| Acupressure for constipation | Ak_Ob | Onkopedia, Kokoninfo, Consumer Advice Centres | Li_Ra |
| Acupressure for heartburn | Ak_Re | S3 Guideline Complementary Medicine in the treatment of oncological patients | Li_S3 |
| Acupressure for hiccups | Ak_Sa | Specific apps for relaxation techniques | Ap_7m |
| Acupressure with Sea-Band | Ak_Se | Links/apps for relaxation techniques | Ap_Ch |
| Acupressure for sleep disorders | Ak_Sf | Autogenic training | Aut |
| Acupressure for pain | Ak_Sz | Hypnotherapy | Hyp |
| Acupressure for nausea | Ak_Ue | MBSR | Mbs |
| Acupressure for digestive problems | Ak_Ve | Meditation | Med |
| Acupressure for cystitis | Ak_Zy | Progressive muscle relaxation | Pmr |
| Acupuncture in general | Ak_Al | Leaflet Psychosocial counselling services | Be_Ps |
| Breathing exercises | At_Ue | Leaflet Acupuncture clinic | Fl_Ak |
| Palm therapy | Pal | Leaflet Hand-foot syndrome | Fl_Ha |
| General advice on complementary medicine | Ber | Referral to outpatient nursing clinic | Fl_Pf |
| CIPN brochure/leaflet | Fl_Ci | Referral to pain centre | Fl_Sz |

### Supplement 2.3: Code sheet Naturopathic Nursing Interventions (NNI)

| **NPI** | **Code** | **NPI** | **Code** |
| --- | --- | --- | --- |
| Eucalyptus oil compress for the lower abdomen | Oe_Eu | Nail taping | Na_Ta |
| Fennel oil compress for the abdomen | Oe_Fe | Nasal care with Coldastop nasal oil | Na_Co |
| Caraway oil compress | Oe_Ku | Nasal douche | Na_Du |
| Lemon balm oil compress | Oe_Me | Seawater nasal spray | Na_Me |
| Cabbage leaf compress | Ko_Au | Nasal care with sesame oil | Na_Se |
| Cottage cheese compress | Qu_Au | Nasal care with shea butter | Na_Sh |
| Partial/full bath with field horsetail tea | Te_Ac | Solum oil compress | Oe_So |
| Partial/full bath with apple cider vinegar | Te_Ap | Aurum Lavendula comp. heart compress | He_Au |
| Partial/full bath with alkaline salt | Te_Ba | Lavender breast oil compress | Oe_La |
| Partial/full bath with baking powder | Te_Bp | Rosemary-orange oil breast compress | Oe_Ro |
| Partial/full bath with lemon balm | Te_Me | Thyme mastichina breast compress | Oe_Th |
| Partial/full bath with Solum | Te_So | Aurum Lavandula comp. forearm rub | Un_Au |
| Partial/full bath with cornflour | Te_Sp | Vagisan cream | Va_Cr |
| Partial/full bath with pansy tea | Te_St | Coconut oil vaginal care | Va_Ko |
| Buttock shower | Po_Du | Majorana vaginal gel | Va_Ma |
| Hip bath with alkaline salt | Si_Ba | Vaginal lactic acid ovules | Va_Mi |
| Hip bath with chamomile | Si_Ka | Vaginal care with shea butter | Va_Sh |
| Hip bath with lavender | Si_La | Dry heat applications (e.g. cherry stone sachets, hot water bottle) | Wa_Tr |
| Hip bath with rose geranium | Si_Ro | Wash with field horsetail tea | Th_Ac |
| Lymphatic drainage oil | Ly_Oe | Therapeutic wash with apple cider vinegar and baking powder | Th_Ap |
| Rosemary foot bath in the morning | Fu_Ro | Wash with alkaline salt | Th_Ba |
| Sage foot bath | Fu_Sa | Therapeutic wash with lavender | Th_La |
| Salt foot bath | Fu_Sz | Therapeutic wash with peppermint hydrosol | Th_Pf |
| Lemon foot wrap or bath | Fu_Zi | Therapeutic wash with rosemary | Th_Ro |
| Hand bath with alkaline salt | Ha_Ba | Therapeutic wash with sage | Th_Sa |
| Hand bath with linseed decoction | Ha_Le | Wash with pansy tea | Th_St |
| Skin care with aloe vera | Ha_Al | Therapeutic wash with lemon | Th_Zi |
| Skin care with Bepanthen | Ha_Be | Inhalation with cajeput | In_Ca |
| Skin care with Coryt Desqua® | Ha_Co | Inhalation with chamomile | In_Ka |
| Skin care with medicinal wool | Ha_He | Inhalation with Plantago bronchial balm | In_Pl |
| Skin care with jojoba oil | Ha_Jo | Inhalation with salt or non-specific | In_Sa |
| Skin care with coconut oil | Ha_Ko | Abdominal rub with caraway oil | Be_Ku |
| Skin care with Linola Radioderm | Ha_Li | Abdominal rub with Wala® lemon balm oil | Be_Me |
| Skin care with almond oil | Ha_Ma | Abdominal rub with Solum oil | Be_So |
| Skin care with menthol cream for CIPN | Ha_Me | Hot compress with chamomile | Fe_Ka |
| Skin care with scar oil | Ha_Na | Hot compress with aniseed, fennel and caraway | Fe_Ku |
| Skin care with Rosatum healing ointment | Ha_Rh | Hot compress with lemon balm | Fe_Me |
| Skin care with castor oil | Ha_Ri | Hot liver compress with yarrow | Fe_Sc |
| Skin care with rose hydrosol | Ha_Ro | Black tea compress | Au_Sw |
| Skin care with sea buckthorn pulp oil | Ha_Sa | Borago essence wrap | Bo_Es |
| Skin care with shea butter | Ha_Sh | Eye drops + Weleda eye drops + Euphrasia | Au_Tr |
| Skin care with urea | Ha_Ur | Fragrance stick (general) | Du_Al |
| Skin care with sugar and oil scrub | Ha_Zu | Fragrance stick (bergamot) | Du_Be |
| Flaxseed decoction infocept | Le_Su | Fragrance stick (lavender) | Du_La |
| Retterspitz wound ointment | Wu_Re | Fragrance stick (orange) | Du_Or |
| Cooling/cold therapy, skin cooling gel pads | Ka_Tr | Fragrance stick (peppermint) | Du_Pf |
| Kneipp treatment | Kn_An | Fragrance stick (rosemary, rosemary/orange, rosemary/lemon) | Du_Ro |
| Contrast showers | We_Gu | Fragrance stick (vanilla) | Du_Va |
| Oral care pineapple juice ice lollies | Mu_An | Fragrance stick (lemon) | Du_Zi |
| Oral care calendula tea | Mu_Ca | Aconite pain relief oil | Ac_Oe |
| Oral care marshmallow root tea | Mu_Ei | Rub (aloe vera gel) | Ei_Al |
| Honey mouthwash | Mu_Ho | Rub (bergamot) | Ei_Be |
| Oral care islamoos | Mu_Is | Calendula oil rub | Ei_Ca |
| Mouthwash chamomile | Mu_Ka | Lavender rub | Ei_La |
| Oral care coconut oil | Mu_Ko | Menthol cream rub | Ei_Me |
| Mouthwash almond-lemon | Mu_Ma | Peppermint oil rub | Ei_Mi |
| Oil pulling | Mu_Oe | Solum oil rub | Ei_So |
| Mouthwash propolis | Mu_Pr | Lavender foot rub | Fe_La |
| Oral are sage tea | Mu_Sa | Rosemary foot rub | Fe_Ro |
| Oral care sea buckthorn pulp oil | Mu_Sd | Solum foot rub | Fe_So |
| Saliva natura | Mu_Sn | General foot bath | Fu_Al |
| Oral care tongue cleaner | Mu_Zu | Alkaline foot bath | Fu_Ba |
| Infocept dysgeusia and taste disturbance | In_Dy | Ginger foot bath | Fu_In |
| Infocept nail changes | In_Na | Lavender foot bath | Fu_La |
| Nail care: alkaline bath | Na_Ba | Flaxseed decoction foot bath | Fu_Le |
| Nail care: healing clay | Na_He | Sage hydrosol for hot flushes | Ei_Sh |
| Nail care: nail hardener | Na_Na | Plantago bronchial balm rub | Ei_Pl |
| Nail care with niaouli oil | Na_Ni |  |  |

### Supplement 2.4: Code sheet Medicaments and Teas (MT)

| **MT** | **Code** | **MT** | **Code** |
| --- | --- | --- | --- |
| Amara | Ama | Dried plums | Pfl |
| Birch charcoal cp | Bir | Sanbitter | San |
| Bolus Alba | Bol | Venoplant | Ven |
| Bryophyllum | Bry | Frankincense | Wei |
| Cardiodoron/Aurum | Car | Hawthorn | Wsd |
| Hepatodoron | Hep | Capsaicin | Cap |
| Mistletoe | Mis | Probiotics | Pro |
| Nux vomica cp | Nux | Selenium | Sel |
| Angocin | Ang | Zinc | Zin |
| Antall | Ant | Laxative tea | Ab_Te |
| Valerian | Bal | Field horsetail tea | Ac_Te |
| Bronchipret | Bro | Stomach tea | Ba_Te |
| Cimicifuga | Cim | Birch leaf tea | Bb_Te |
| Cranberry | Cra | Bitter tea | Bi_Te |
| Cystinol | Cys | Nettle tea | Br_Te |
| Gentiana lutea | Enz | Dill seeds tea | Di_Te |
| Psyllium husks | Flo | Fennel seeds tea | Fe_Te |
| Gelomyrtol | Gel | Green tea | Gr_Te |
| Ginseng | Gin | Blueberry tea | He_Te |
| Ipalat | Ipa | Ginger tea | In_Te |
| Pumpkin seed capsules | Kue | Chamomile tea | Ka_Te |
| Lasea | Las | Peppermint tea | Pf_Te |
| Linseed | Lei | Sage tea | Sa_Te |
| Mannose | Man | Sleep tea | Sf_Te |
| Milk thistle | Mar | Yarrow tea | Sg_Te |
| Cranberry | Moo | Pansies tea | St_Te |
| Myrrhinil intest | Myr | Black tea | Sw_Te |
| Neurexan | Neu | Thyme tea | Th_Te |
| Peppermint oil capsules | Pfe |  |  |
