# Supplementary material for: Reasons for encounter and recommended interventions in an interprofessional integrative oncology counseling program in outpatient care: a mixed-methods analysis of 1,693 consultation letters
Source: Front Med (Lausanne). 2026 Oct 7;13:1907654. doi: 10.3389/fmed.2026.1907654 (PMC13683791; doi:10.3389/fmed.2026.1907654)
Supplement: Supplementary file 3 [file Table3.docx]

|  | **Total** | **%** | **Curative** | **%** | **Palliative** | **%** | **Unclear** | **%** |
| --- | --- | --- | --- | --- | --- | --- | --- | --- |
| **Reason for Encounter** | n = 603 | 100% | n = 320 | 53% | n = 202 | 33% | n = 81 | 13% |
| **Cancer-related Fatigue** | 336 | 56% | 176 | 55% | 119 | 59% | 41 | 51% |
| **General questions about CIH** | 264 | 44% | 141 | 44% | 92 | 46% | 31 | 38% |
| **CIPN** | 244 | 40% | 139 | 43% | 75 | 37% | 30 | 37% |
| **Sleep disturbances** | 226 | 37% | 126 | 39% | 72 | 36% | 28 | 35% |
| **Pain** | 215 | 36% | 102 | 32% | 79 | 39% | 34 | 42% |
| **Skin reactions** | 199 | 33% | 130 | 41% | 43 | 21% | 26 | 32% |
| **Mental stress** | 195 | 32% | 85 | 27% | 78 | 39% | 32 | 40% |
| **Nausea** | 193 | 32% | 103 | 32% | 56 | 28% | 34 | 42% |
| **Mucositis and Xerostomia** | 184 | 31% | 102 | 32% | 59 | 29% | 23 | 28% |
| **Anxiety/restlessness** | 153 | 25% | 86 | 27% | 56 | 28% | 11 | 14% |
| **General digestive complaints** | 149 | 25% | 78 | 24% | 55 | 27% | 16 | 20% |
| **Dysgeusia** | 138 | 23% | 80 | 25% | 44 | 22% | 14 | 17% |
| **General mucosal problems** | 127 | 21% | 64 | 20% | 49 | 24% | 14 | 17% |
| **Diarrhea** | 99 | 16% | 51 | 16% | 35 | 17% | 13 | 16% |
| **Loss of appetite** | 96 | 16% | 38 | 12% | 49 | 24% | 9 | 11% |
| **Constipation** | 95 | 16% | 47 | 15% | 36 | 18% | 12 | 15% |
| **Nail changes** | 78 | 13% | 55 | 17% | 15 | 7% | 8 | 10% |
| **Menopausal symptoms (hot flashes)** | 75 | 12% | 52 | 16% | 17 | 8% | 6 | 7% |
| **Lymphedema** | 64 | 11% | 41 | 13% | 19 | 9% | 4 | 5% |
| **Cold/heat sensitivity** | 63 | 10% | 42 | 13% | 10 | 5% | 11 | 14% |
| **Hand-foot syndrome** | 41 | 7% | 17 | 5% | 19 | 9% | 5 | 6% |

**Specific Reasons for Encounter by Treatment Intent**
