# Supplementary material for: Reasons for encounter and recommended interventions in an interprofessional integrative oncology counseling program in outpatient care: a mixed-methods analysis of 1,693 consultation letters
Source: Front Med (Lausanne). 2026 Oct 7;13:1907654. doi: 10.3389/fmed.2026.1907654 (PMC13683791; doi:10.3389/fmed.2026.1907654)
Supplement: Supplementary file 4 [file Table4.docx]

|  | **Total** | **%** | **Curative** | **%** | **Palliative** | **%** | **Unclear** | **%** |
| --- | --- | --- | --- | --- | --- | --- | --- | --- |
|  | **n = 603** | **100%** | **n = 320** | **53%** | **n = 202** | **33%** | **n = 81** | **13%** |
| **Lifestyle Modification** |  |  |  |  |  |  |  |  |
| Nutritional counseling | 570 | 95% | 304 | 95% | 190 | 94% | 76 | 94% |
| Physical activity | 558 | 93% | 300 | 94% | 183 | 91% | 75 | 93% |
| Relaxation strategies | 487 | 81% | 267 | 83% | 148 | 73% | 72 | 89% |
| **Non-Pharmacological Interventions** | **588** | **98%** | **311** | **97%** | **198** | **98%** | **79** | **98%** |
| Acupressure | 475 | 79% | 248 | 78% | 162 | 80% | 65 | 80% |
| Referrals and Specific Guides | 438 | 73% | 224 | 70% | 154 | 76% | 60 | 74% |
| Specific Nutritional Counseling | 369 | 61% | 188 | 59% | 137 | 68% | 44 | 54% |
| Specific Exercise Counseling | 321 | 53% | 158 | 49% | 120 | 59% | 43 | 53% |
| Mindfulness-Based Movement Therapies | 264 | 44% | 135 | 42% | 88 | 44% | 41 | 51% |
| Instruction in Relaxation Techniques | 175 | 29% | 91 | 28% | 59 | 29% | 25 | 31% |
| **Naturopathic Nursing Interventions** | **582** | **97%** | **311** | **97%** | **193** | **96%** | **78** | **96%** |
| Skin Care | 343 | 57% | 188 | 59% | 110 | 54% | 45 | 56% |
| Rubdowns | 334 | 55% | 169 | 53% | 121 | 60% | 43 | 53% |
| Partial/Full Baths | 308 | 51% | 166 | 52% | 92 | 46% | 46 | 57% |
| Oils and Ointment Compresses | 305 | 51% | 170 | 53% | 107 | 53% | 31 | 38% |
| Inhalations | 280 | 46% | 138 | 43% | 109 | 54% | 33 | 41% |
| Mucosal Care | 241 | 40% | 121 | 38% | 80 | 40% | 28 | 35% |
| Moist Compresses and Wraps | 230 | 38% | 130 | 41% | 82 | 41% | 29 | 36% |
| Therapeutic Washing | 121 | 20% | 70 | 22% | 41 | 20% | 10 | 12% |
| Cold and Heat Applications | 121 | 20% | 66 | 21% | 39 | 19% | 15 | 19% |
| **Medicines and Teas** | **475** | **79%** | **248** | **78%** | **168** | **83%** | **59** | **73%** |
| Medicinal Teas | 355 | 59% | 181 | 57% | 132 | 65% | 42 | 52% |
| Phytotherapeutics | 330 | 55% | 171 | 53% | 117 | 58% | 41 | 51% |
| Anthroposophic Remedies | 61 | 10% | 43 | 13% | 10 | 5% | 8 | 10% |

**Lifestyle Modification and Specific CIH Interventions by Treatment Intent**
