# Supplementary material for: Reasons for encounter and recommended interventions in an interprofessional integrative oncology counseling program in outpatient care: a mixed-methods analysis of 1,693 consultation letters
Source: Front Med (Lausanne). 2026 Oct 7;13:1907654. doi: 10.3389/fmed.2026.1907654 (PMC13683791; doi:10.3389/fmed.2026.1907654)
Supplement: Supplementary file 5 [file Table5.docx]

|  | **Total** | **%** | **Breast  Cancer** | **%** | **Gastrointestinal  Cancer** | **%** | **Gyneological  Cancer** | **%** | **Respiratory**  **Cancer** |  | **Other** | **%** |
| --- | --- | --- | --- | --- | --- | --- | --- | --- | --- | --- | --- | --- |
| **Reason for Encounter** | n = 603 | 100% | n = 276 | 46% | n = 105 | 17% | n = 50 | 8% | n = 36 | 6% | n = 136 | 23% |
| **Cancer-related Fatigue** | 336 | 56% | 169 | 61% | 59 | 56% | 24 | 48% | 22 | 61% | 62 | 46% |
| **General questions about CIH** | 264 | 44% | 131 | 47% | 51 | 49% | 16 | 32% | 16 | 44% | 50 | 37% |
| **CIPN** | 244 | 40% | 131 | 47% | 57 | 54% | 24 | 48% | 4 | 11% | 28 | 21% |
| **Sleep disturbances** | 226 | 37% | 120 | 43% | 29 | 28% | 18 | 36% | 12 | 33% | 47 | 35% |
| **Pain** | 215 | 36% | 101 | 37% | 25 | 24% | 24 | 48% | 11 | 31% | 54 | 40% |
| **Skin reactions** | 199 | 33% | 123 | 45% | 19 | 18% | 15 | 30% | 8 | 22% | 34 | 25% |
| **Mental stress** | 195 | 32% | 85 | 31% | 38 | 36% | 19 | 38% | 15 | 42% | 38 | 28% |
| **Nausea** | 193 | 32% | 93 | 34% | 38 | 36% | 17 | 34% | 6 | 17% | 39 | 29% |
| **Mucositis and Xerostomia** | 184 | 31% | 102 | 37% | 31 | 30% | 9 | 18% | 8 | 22% | 34 | 25% |
| **Anxiety/restlessness** | 153 | 25% | 74 | 27% | 19 | 18% | 12 | 24% | 10 | 28% | 38 | 28% |
| **General digestive complaints** | 149 | 25% | 52 | 19% | 38 | 36% | 19 | 38% | 8 | 22% | 32 | 24% |
| **Dysgeusia** | 138 | 23% | 76 | 28% | 24 | 23% | 11 | 22% | 7 | 19% | 20 | 15% |
| **General mucosal problems** | 127 | 21% | 67 | 24% | 25 | 24% | 13 | 26% | 8 | 22% | 14 | 10% |
| **Diarrhea** | 99 | 16% | 45 | 16% | 27 | 26% | 8 | 16% | 3 | 8% | 16 | 12% |
| **Loss of appetite** | 96 | 16% | 32 | 12% | 31 | 30% | 7 | 14% | 9 | 25% | 17 | 13% |
| **Constipation** | 95 | 16% | 43 | 16% | 18 | 17% | 10 | 20% | 6 | 17% | 18 | 13% |
| **Nail changes** | 78 | 13% | 57 | 21% | 4 | 4% | 4 | 8% | 4 | 11% | 9 | 7% |
| **Menopausal symptoms (hot flashes)** | 75 | 12% | 51 | 18% | 6 | 6% | 4 | 8% | 2 | 6% | 12 | 9% |
| **Lymphedema** | 64 | 11% | 36 | 13% | 5 | 5% | 9 | 18% | 0 | 0% | 14 | 10% |
| **Cold/heat sensitivity** | 63 | 10% | 32 | 12% | 9 | 9% | 6 | 12% | 1 | 3% | 15 | 11% |
| **Hand-foot syndrome** | 41 | 7% | 27 | 10% | 10 | 10% | 0 | 0% | 1 | 3% | 3 | 2% |

**Specific Reasons for Encounter by Cancer Diagnosis**
