# Supplementary material for: Reasons for encounter and recommended interventions in an interprofessional integrative oncology counseling program in outpatient care: a mixed-methods analysis of 1,693 consultation letters
Source: Front Med (Lausanne). 2026 Oct 7;13:1907654. doi: 10.3389/fmed.2026.1907654 (PMC13683791; doi:10.3389/fmed.2026.1907654)
Supplement: Supplementary file 6 [file Table6.docx]

|  | **Total** | **%** | **Breast  Cancer** | **%** | **Gastrointestinal  Cancer** | **%** | **Gyneological  Cancer** | **%** | **Respiratory  Cancer** |  | **Other** | **%** |
| --- | --- | --- | --- | --- | --- | --- | --- | --- | --- | --- | --- | --- |
|  | n = 603 | 100% | n = 276 | 46% | n = 105 | 17% | n = 50 | 8% | n = 36 | 6% | n = 136 | 23% |
| **Lifestyle Modification** |  |  |  |  |  |  |  |  |  |  |  |  |
| Nutritional counseling | 570 | 95% | 263 | 95% | 94 | 90% | 49 | 98% | 35 | 97% | 129 | 95% |
| Physical activity | 558 | 93% | 256 | 93% | 94 | 90% | 47 | 94% | 34 | 94% | 127 | 93% |
| Relaxation strategies | 487 | 81% | 225 | 82% | 70 | 67% | 45 | 90% | 29 | 81% | 118 | 87% |
| **Non-Pharmacological Interventions** | **588** | **98%** | **272** | **99%** | **100** | **95%** | **49** | **98%** | **36** | **100%** | **131** | **96%** |
| Acupressure | 475 | 79% | 225 | 82% | 85 | 81% | 42 | 84% | 26 | 72% | 97 | 71% |
| Referrals and Specific Guides | 438 | 73% | 212 | 77% | 74 | 70% | 34 | 68% | 26 | 72% | 92 | 68% |
| Specific Nutritional Counseling | 369 | 61% | 166 | 60% | 73 | 70% | 33 | 66% | 16 | 44% | 81 | 60% |
| Specific Exercise Counseling | 321 | 53% | 152 | 55% | 54 | 51% | 24 | 48% | 22 | 61% | 69 | 51% |
| Mindfulness-Based Movement Therapies | 264 | 44% | 119 | 43% | 48 | 46% | 20 | 40% | 22 | 61% | 55 | 40% |
| Instruction in Relaxation Techniques | 175 | 29% | 77 | 28% | 24 | 23% | 17 | 34% | 14 | 39% | 43 | 32% |
| **Naturopathic Nursing Interventions** | **582** | **97%** | **270** | **98%** | **97** | **92%** | **50** | **100%** | **34** | **94%** | **131** | **96%** |
| Skin Care | 343 | 57% | 184 | 67% | 57 | 54% | 30 | 60% | 14 | 39% | 58 | 43% |
| Rubdowns | 334 | 55% | 152 | 55% | 58 | 55% | 28 | 56% | 18 | 50% | 77 | 57% |
| Partial/Full Baths | 308 | 51% | 146 | 53% | 38 | 36% | 31 | 62% | 17 | 47% | 72 | 53% |
| Oils and Ointment Compresses | 305 | 51% | 153 | 55% | 44 | 42% | 32 | 64% | 20 | 56% | 59 | 43% |
| Inhalations | 280 | 46% | 134 | 49% | 46 | 44% | 22 | 44% | 16 | 44% | 62 | 46% |
| Mucosal Care | 241 | 40% | 117 | 42% | 43 | 41% | 15 | 30% | 14 | 39% | 40 | 29% |
| Moist Compresses and Wraps | 230 | 38% | 123 | 45% | 36 | 34% | 24 | 48% | 15 | 42% | 43 | 32% |
| Therapeutic Washing | 121 | 20% | 68 | 25% | 16 | 15% | 6 | 12% | 7 | 19% | 24 | 18% |
| Cold and Heat Applications | 121 | 20% | 58 | 21% | 18 | 17% | 15 | 30% | 7 | 19% | 22 | 16% |
| **Medicines and Teas** | **475** | **79%** | **219** | **79%** | **83** | **79%** | **46** | **92%** | **29** | **81%** | **98** | **72%** |
| Medicinal Teas | 355 | 59% | 162 | 59% | 69 | 66% | 35 | 70% | 24 | 67% | 65 | 48% |
| Phytotherapeutics | 330 | 55% | 157 | 57% | 61 | 58% | 27 | 54% | 22 | 61% | 62 | 46% |
| Anthroposophic Remedies | 61 | 10% | 31 | 11% | 9 | 9% | 5 | 10% | 2 | 6% | 14 | 10% |

**Lifestyle Modification and specific CIH Interventions by Cancer Diagnosis**
